# Transcriptomic and Genetic Profiling of HIV-Associated Neurocognitive Disorders

**DOI:** 10.3389/fmolb.2021.721954

**Published:** 2021-10-29

**Authors:** Daniel Ojeda-Juárez, Marcus Kaul

**Affiliations:** Division of Biomedical Sciences, School of Medicine, University of California, Riverside, Riverside, CA, United States

**Keywords:** HIV, HIV-associated neurocognitive disorders, neurodegeneration, transcriptomics, genome-wide association, epigenetics, genomics

## Abstract

Early in the HIV pandemic, it became evident that people living with HIV (PLWH) develop a wide range of neurological and neurocognitive complications. Even after the introduction of combination antiretroviral therapy (cART), which dramatically improved survival of PLWH, the overall number of people living with some form of HIV-associated neurocognitive disorders (HAND) seemed to remain unchanged, although the incidence of dementia declined and questions about the incidence and diagnosis of the mildest form of HAND arose. To better understand this complex disease, several transcriptomic analyses have been conducted in autopsy samples, as well as in non-human primates and small animal rodent models. However, genetic studies in the HIV field have mostly focused on the genetic makeup of the immune system. Much less is known about the genetic underpinnings of HAND. Here, we provide a summary of reported transcriptomic and epigenetic changes in HAND, as well as some of the potential genetic underpinnings that have been linked to HAND, and discuss future directions with hurdles to overcome and angles that remain to be explored.

## Introduction

Human immunodeficiency virus-1 (HIV-1) is a lentivirus of the Retroviridae family, and it is the causative agent of the acquired immunodeficiency syndrome (AIDS) (Barre-Sinoussi et al., 1983; Gallo et al., 1984; Hahn et al., 1984; Navia et al., 1986a). HIV-1 infection remains a serious threat to global health, with around 38 million people living with HIV-1 (PLWH) as of 2020 (Global HIV and AIDS statistics, 2020). The introduction of combination antiretroviral therapy (cART) decreased morbidity and mortality associated with HIV infection by effectively suppressing viral replication, and it changed HIV-1 infection from a fatal to a chronic manageable disease (Palella et al., 1998; McArthur et al., 2010; Heaton et al., 2011; Vella et al., 2012; Saylor et al., 2016).

Early in the HIV pandemic before anti-retroviral treatments were available, it became evident that HIV-infection can cause neurological and neurocognitive complications that were referred to as AIDS dementia complex (ADC) and ranged from minor cognitive and motor disorder (MCMD) to full-blown HIV-associated dementia (HAD) (Loewenstein and Sharfstein, 1983; Navia et al., 1986b). At the same time autopsies of many patients who had died from AIDS showed a pronounced neuropathology that is referred to as HIV encephalitis (HIVE) and was often interpreted as the histopathological correlate of ADC, although it was noted that not all HAD cases displayed HIVE (Navia et al., 1986a). Several tools were developed to classify the neurological and neurocognitive complications of HIV infection, including the Memorial Sloan Kettering classification of HAD (MSK) (Price and Brew, 1988; Tross et al., 1988), the criteria of the American Academy of Neurology (AAN) (listed, 1991), and the HIV dementia scale (Power et al., 1995). However, the introduction of cART reshaped the HIV epidemic, including the incidence of neurological, neurocognitive and neuropsychiatric complications, which were therefore collectively reclassified in 2007 as HIV-associated neurocognitive disorders (HAND) using what is often referred to as the Frascati criteria (Antinori et al., 2007). HAND categories range from Asymptomatic Neurocognitive Impairment (ANI) to Mild Neurocognitive Disorder (MND) to full-blown HIV-associated dementia (HAD) (Antinori et al., 2007). By definition, HAND is caused by HIV-1, not an opportunistic infection or other brain disease associated with the viral infection. The criteria for a diagnosis of HAND were based on the poor performance on neurocognitive tests when compared to a control population that has to be matched for a number of parameters, in particular age, gender, ethnicity and years of education, and the criteria were intended for research (Antinori et al., 2007).

Despite viral suppression, PLWH continue to develop neurocognitive complications that fall into the HAND classifications (McArthur et al., 2010; Joseph et al., 2016; Saylor et al., 2016). The introduction of cART resulted in a shift from more severe forms of HAND towards less severe forms, with ANI becoming the most common type of HAND in the post-cART era (McArthur et al., 2010; Heaton et al., 2011; Saylor et al., 2016). However, the overall percentage of patients with HAND appeared not significantly reduced (McArthur et al., 2010; Heaton et al., 2011; Saylor et al., 2016). A recent meta-analysis of 35,513 individuals from 123 studies conducted in 32 countries identified a world-wide prevalence of 42.6% for HAND with an estimated 72% of cases occurring in sub-Saharan Africa (Wang et al., 2020). However, the attempt to widely use the current classification to assess the clinical burden of HAND has revealed a number of challenges regarding the diagnosis (Nightingale et al., 2021). One is the standardization and comparability of assessments across different observers administering the tests, different languages, ethnicities, and cultural and social backgrounds (Woods et al., 2004). Another challenge is the fact that performance on neurocognitive tests can be affected by a variety of factors besides HIV infection and HIVE, such as brain pathology due to cerebrovascular disease, other subclinical co-morbidities, lifestyle and complex social factors beyond education (Nightingale et al., 2021). Thus, it may not be surprising that over the years the clinical relevance of HAND, and particularly the diagnosis of ANI, has become a matter of some debate as many individuals classified as having ANI show no evidence of limitations in everyday functioning (Antinori et al., 2007; Saloner and Cysique, 2017; Ciccarelli, 2020; Nightingale et al., 2021). On the other hand, a CNS HIV Antiretroviral Therapy Effects Research (CHARTER) study found that participants who had ANI at baseline had a 2- to 6-fold increased risk for earlier development of symptomatic HAND than neurocognitive normal individuals (Grant et al., 2014). Moreover, a recent study (Neuro+3) indicated that an intensification of cART with increased CNS penetration effectiveness (CPE) scores can improve neurocognitive performance of HIV^+^ individuals, including those with ANI diagnosis (Force et al., 2021). In response to the challenges associated with HAND diagnoses, numerous studies that will be reviewed below have used combinations of scores of neurocognitive and neuropsychiatric assessments in order to establish for example a global deficit score (GDS) rather than placing an individual into a HAND category. Some studies have also combined GDS and HAND classification. Besides a call for better international standardization, various modifications have been proposed for a better consensus on the diagnosis of neurocognitive impairment in PLWH, such as an adjustment of the limit score used to distinguish ANI from normal performance, treating cognitive performance as a continuous variable, and including clinical history in the diagnosis (Saloner and Cysique, 2017; Ciccarelli, 2020; Nightingale et al., 2021). In any case, another challenge that remains for research is the comparison of neurocognitive and neuropsychiatric assessments made before and after the introduction of cART and before and after the establishment of HAND based on the Frascati criteria. Development of HIV-associated neurocognitive complications in the cART era may be driven by a combination of factors, such as small amounts of HIV replication in the central nervous system (CNS), viral proteins, neuropsychiatric adverse effects of cART, or substance abuse at any age, medical co-morbidities, such as coinfections with hepatitis C, cardiovascular disease and diabetes, aging, gender, and sociodemographic setting (Becker et al., 2009; Foley et al., 2010; Wright et al., 2010; Crystal et al., 2012; Ciccarelli et al., 2013; DeVaughn et al., 2015; Sanchez et al., 2016a; Sanchez and Kaul, 2017; Saloner et al., 2019; Yuan and Kaul, 2021). The specific contributions of each of these factors in the pathogenesis of HAND are still incompletely understood.

The usage of transcriptomic and genetic techniques, such as microarrays and DNA- and RNA-sequencing (RNA-seq) to study genome-wide variants and changes in gene expression are powerful tools to characterize and discover mechanisms leading to the development of neurocognitive deficits and neuropathology in PLWH. However, few studies employing these technologies have investigated human brain samples of individuals with HAND. Surprisingly, only eight microarray studies (Gelman et al., 2004; Masliah et al., 2004; Shapshak et al., 2004; Everall et al., 2005; Borjabad et al., 2011; Gelman et al., 2012; Zhou et al., 2012; Levine et al., 2013) and a single report of RNA-seq (Canchi et al., 2020) analyzed brain tissues of HIV^+^ individuals (Tables 1, 2). Of seven epigenetic studies analyzing *post-mortem* brain tissues of HIV^+^ individuals, only four specifically addressed HAND (Yelamanchili et al., 2010; Zhou et al., 2012; Levine et al., 2016; Xu et al., 2017) (part of Table 3). Of 40 genetic studies testing association of candidate genes with HIV-associated neurocognitive impairment, neuropsychiatric problems or HAND, only eight specifically stated that they analyzed brain tissues (Dunlop et al., 1997; Sato-Matsumura et al., 1998; Boven et al., 1999; Quasney et al., 2001; Cutler et al., 2004; Diaz-Arrastia et al., 2004; Levine et al., 2009; Soontornniyomkij et al., 2012) while most used peripheral blood mononuclear cells (PBMC), whole blood or other tissues (Table 4). Four genome-wide association studies have addressed HAND but only three specified the cell types used, which were PBMC or whole blood (Levine et al., 2012a; van Manen et al., 2012; Jia et al., 2017; Hulgan et al., 2019) (Table 4). Some of the transcriptomic and genetic studies identified genes that correlated with neuropathological signs of HIV infection or HIVE which, however, do not always correlate with neurocognitive changes. Nonetheless, the combined data of all the studies still provide inroads to discovery, comparison to other neurodegenerative diseases, and opportunities for cross-validation with animal and cellular models for the study of HAND. Additionally, analyzing the changes in the transcriptome of animal models can in turn lead to the discovery of new mechanisms or molecules driving neurocognitive impairment in PLWH.

**TABLE 1:** Published Gene Expression Studies. List of the eight microarray, and one RNA-seq studies performed using *post-mortem* brain tissues of HIV-infected and uninfected individuals. AD, Alzheimer’s Disease; HIV^−^/Ctl, uninfected, healthy control; HIVE, HIVE encephalitis; HAND, HIV associated neurocognitive disorders; NCI, Neurocognitive impairment; HAD, HIV-associated dementia; HIV+/CN, HIV+ cognitive normal subjects; NPI-O, neuropsychologically impaired but not definitely caused by HIV infection due to comorbid factor; MCMD, minor cognitive and motor disorder; MND, Mild neurocognitive disorder; METH, methamphetamine; WGCNA, weighted gene co-expression network analysis.

Study	Groups included in the study (number of cases)	Area(s) of the brain	Method	References
Masliah et al. (2004)	HIV+ with HIVE (*n* = 9) vs. HIV+ no HIVE (*n* = 8)	Frontal Cortex	Microarray	Masliah et al. (2004)
Gelman et al. (2004)	HIV+ with HAD (*n* = 5) or MCMD (*n* = 2) or NPI-O (*n* = 2) vs. HIV-/Ctl (*n* = 5)	Frontal cortical grey matter and adjacent gyral white matter	Microarray	Gelman et al. (2004)
Shapshak et al. (2004)	HIV+ (*n* = 5 incl. HIVE (*n* = 3) and/or HAD (*n* = 3)) vs. HIV-/Ctl (*n* = 4)	Frontal cortex	Microarray	Shapshak et al. (2004)
Everall et al. (2005)	HIV+ HIVE/METH (*n* = 4) vs. HIV+ HIVE, no METH (*n* = 4) vs. HIV+ no HIVE, no METH (*n* = 5)	Frontal cortex	Microarray	Everall et al. (2005)
Borjabad et al. (2011)	HIV+ HAND (*n* = 8) vs. HIV+ cART, HAND (*n* = 7) vs. HIV−/Ctl (*n* = 6)	Deep white matter within anterior frontal lobe	Microarray	Borjabad et al. (2011)
Gelman et al. (2012)	HIV+ with NCI and HIVE (*n* = 5) vs. HIV+ with NCI, no HIVE (*n* = 7) vs. HIV+/CN, no HIVE (*n* = 6) vs. HIV−/Ctl (*n* = 6)	Frontal cortex, neostriatum, and white matter from deep frontal lobe	Microarray	Gelman et al. (2012)
Zhou et al. (2012)	HIV+ with HAD (*n* = 10) vs. HIV+ no HAD (*n* = 8)	Frontal Cortex	Microarray	Zhou et al. (2012)
Levine et al. (2013)	HIV+ with HAD and HIVE (*n* = 4) vs. HIV+ with HAD, no HIVE (*n* = 7) vs. HIV+/CN, no HIVE (*n* = 4) vs. HIV−/Ctl (*n* = 6 (microarray), *n* = 8 (WGCNA)) vs. AD+ HIV− (*n* = 20)	Frontal cortex, neo-striatum, and white matter from deep frontal lobe (see Gelman et al. (2012))	Microarray and WGCNA	Levine et al. (2013)
Canchi et al. (2020)	HIV+ with HAD (*n* = 3) vs. HIV+ with MND (*n* = 10) vs. HIV+ with ANI (*n* = 10) vs. HIV+/CN (*n* = 10)	Frontal Cortex	RNA-seq	Canchi et al. (2020)
Total *n* in trans-scriptomic studies	HIV+ with or without NCI or HIVE (*n* = 143) vs. HIV−/Ctl (*n* = 29)			

**TABLE 2:** Top Predicted Networks Altered Based on Transcriptomic Changes. * No canonical pathways altered but genes significantly altered were characteristically expressed on microvascular endothelial cells, and perivascular cells. # Study specifically looked at ion channel-related genes. @ GAzar software was used to identify top GO pathways affected. AD, Alzheimer’s Disease; MS, Multiple Sclerosis; ART, antiretroviral therapy. ^$^ IPA was used to predict top canonical pathways altered. ^&^ Gene categories designated by authors, no directionality was provided. *Genes altered were correlated with NCI. to identify common impairment genes. Common genes used for enrichment analysis.

Study	Groups included in the study	Networks upregulated	Networks downregulated
Masliah et al. (2004)	HIV+ with HIVE vs. HIV+ no HIVE	Interferon/antiviral response, neuroinflammation, cell cycle, transcription, signaling, cytoskeletal proteins, cell adhesion/extracellular matrix	Signaling, neuronal, ion transport, cell cycle, transcriptional regulators, cytoskeletal proteins, cell adhesion/extracellular matrix
Gelman et al. (2004)	HIV− vs. HIV+ with HAD or MCMD or NPI-O	Ion transport^#^	Ion transport^#^
Shapshak et al. (2004)	HIV+ incl. HIVE and/or HAD vs. HIV—/Ctl	Signaling, splicing, cell cycle, NMDA receptor signaling, synapse, protein tags, ribosomal proteins, transcription, senescence, proteasome, cytoskeleton, ion transport, mitochondrial translation^&^	
Everall et al. (2005)	HIV+ no HIVE vs. HIV+ HIVE vs. HIV+ HIVE plus METH	Signaling, ion transport, interferon/antiviral response, cell adhesion/extracellular matrix, neuroinflammation, cell cycle, transcription, cytoskeletal proteins	Signaling, ion transport, neuronal, cell adhesion/extracellular matrix, DNA repair, cell cycle, transcription, cytoskeletal proteins
Borjabad et al. (2011)	HIV- vs. untreated HAND with and without HIVE	Immune response, antigen presentation, endogenous antigen, antigen processing, endogenous antigen via MHC class I, inflammatory response, response to virus, antigen presentation, exogenous antigen, antigen processing, exogenous antigen via MHC class II, complement activation, classical pathway^@^	Synaptic transmission, neurogenesis, homophilic cell adhesion, potassium ion transport, oxygen transport, ion transport, cation transport, sodium ion transport^@^
	Untreated HAND vs. cART treated HAND	Immune response, interferon response, regulation of cell cycle	Myelin-related, microtubule-based movement, regulation of cell cycle
Borjabad and Volsky, (2012)	HAND from groups in five HIV studies Masliah et al. (2004); Gelman et al. (2004); Shapshak et al. (2004); Everall et al. (2005); Borjabad et al. (2011) vs. 18 AD	Immune response, defense response, response to biotic stimulus, response to external stimulus, antigen presentation	Transmission of nerve impulse, synaptic transmission, transport, cell-cell signaling, synaptic vesicle
	ART-HAND from groups in five HIV studies vs. 18 AD	Immune response, defense response, antigen processing, antigen presentation, signal transducer activity	DNA helicase activity, hydrolase activity, cell growth and/or maintenance, binding
	HAND from groups in five HIV studies vs. nine MS studies	Immune response, defense response, response to biotic stimulus, response to external stimulus, response stress	Calmodulin-dependent protein kinase I activity, non-kinase phorbol ester receptor activity, calcium/calmodulin-dependent kinase activity, phorbol ester receptor activity
	ART-HAND from groups in five HIV studies vs. nine MS studies	Immune response, defense response, antigen processing, response to biotic stimulus, response to external stimulus	Intracellular signaling cascade, signal transduction, catalytic activity, cell communication, cellular process
Gelman et al. (2012)	HIV−/Ctl vs. HIV+/CN with no HIVE	—	G-coupled receptor signaling, cyclic AMP-mediated signaling^$^
	HIV−/Ctl vs. HIV+ with NCI and no HIVE	—	Alterations in brain microvascular endothelial cells, and perivascular cells*
	HIV−/Ctl vs. HIV+ with NCI and HIVE	Interferon/antiviral response, activation of IRF cytosolic pattern recognition receptor, antigen presentation, pattern recognition receptors for bacteria and viruses, complement system, acute phase response signaling, hepatic fibrosis, toll-like receptor signaling, protein ubiquitination pathway, caveolar mediated endocytosis, liver X receptor^$^	GABA receptor signaling, clathrin mediated endocytosis, glutamate receptor signaling, 14-3-3 mediated signaling, synaptic long-term potentiation, cyclic AMP-mediated signaling, calcium signaling, axonal guidance signaling, chemokine signaling, ephrin receptor signaling, oxidative phosphorylation^$^
Zhou et al. (2012)	HIV+ HAD vs. HIV+ no dementia		Dendrite, ATPase activity coupled to transmembrane movement of ions, ATPase activity coupled to transmembrane movement of ions phosphorylation mechanism, regulation of blood pressure, protein homo oligomerization, mRNA binding, feeding behavior, and cytoskeleton dependent intracellular transport
Levine et al. (2013)	HIV+ with HAD and HIVE vs. HIV+ with HAD, no HIVE vs. HIV+/CN, no HIVE*	Cortex: Transcription regulation, receptor activity, DNA binding. NCI basal ganglia: protein kinase activity, Homo Sapiens 19	Cortex: Mitochondrion, mitotic cell cycle, DNA repair, proteasome complex, nucleus
		White matter: transcription regulation, Homo Sapiens 19, chondroitin/heparin sulfate biosynthesis	
	HIV+ with HAD and HIVE, HIV+ with HAD, no HIVE, HIV+/CN, no HIVE vs. HIV−/Ctl vs. AD+ HIV−*	Cell differentiation, activator, repeat, cell communication, regulation of transcription, phosphorylation	Cytoplasm, energy pathways, mitochondrion, tricarboxylic acid cycle, transit peptide, synaptic vesicle
Canchi et al. (2020)	HIV+ with HAD vs. HIV+ with MND vs. HIV+ with ANI vs. HIV+/CN	Immune response, RNA editing	Synaptic maintenance

**TABLE 3:** Epigenetics Studies. List of studies performed using postmortem brain tissues or blood-derived cells of HIV-infected and uninfected individuals and specific cell types. AD, Alzheimer’s Disease; DTI, diffusion tensor imaging (neuroimaging); HIV^−^/Ctl, uninfected, healthy control; HIVE, HIVE encephalitis; HAND, HIV associated neurocognitive disorders; NCI, Neurocognitive impairment; HAD, HIV-associated dementia; HIV+/CN, HIV+ cognitive normal subjects; NPI-O, neuropsychologically impaired but not definitely caused by HIV infection due to comorbid factor; MCMD, minor cognitive and motor disorder; MND, Mild neurocognitive disorder; MDD, major depressive disorder; METH, methamphetamine; MRI, magnetic resonance imaging (neuroimaging); NHP, non-human primate; qRT-PCR, quantitative reverse transcription polymerase chain reaction.

Study (References)	Groups included in the study (number of cases)	Area(s) of the brain	Method	Notes, or miR/target
*In vitro* cultures				
Eletto et al. (2008)	*In vitro*, HIV Tat vs. untreated	Primary rat cortical neuronal cell cultures	miRNA microarray	miR-128a/SNAP25
Mukerjee et al. (2011)	*In vitro*, HIV Vpr vs. untreated	Primary neuronal cell cultures (human) and SHSY-5Y cell line	miR and mRNA Microarray	Multiple miRs/multiple neuronal genes
Dave and Khalili (2010)	*In vitro*, morphine treated vs. untreated, HIV+ vs. uninfected	Human monocyte-derived macrophages	miRNA microarray	26 miRs/FGF-2, MCP-2, IL-6
Saiyed et al. (2011)	*In vitro*, HIV Tat vs. untreated with and without siRNA for knockdown of HDAC2	Primary neuronal cell cultures (human) and SK-N-MC cell line	HDAC knockdown and qRT-PCR	HDAC reduces CREB and CaMKIIa expr
*Post-mortem* brain tissue				
Yelamanchili et al. (2010)	HIV+ with HAD and HIVE (*n* = 3) vs. HIV+ no HAD, no HIVE (*n* = 4) (NHP: SIV+ with SIVE (*n* = 4) vs. uninfected (*n* = 4))	Caudate nucleus (striatum) of HAD and HIVE, and hippo-campus and striatum of SIV+ rhesus monkeys	miRNA microarray, miR qRT-PCR	miR-21/MEF2C
Noorbakhsh et al. (2010)	HIV+ with HIVE (*n* = 4) vs. HIV−/Ctl (*n* = 4)	Frontal lobe (cortex)	miRNA microarray, qRT-PCR	Multiple miRs/inflammation, immune, cell death genes
Tatro et al. (2010)	HIV+ with MDD (*n* = 5) vs. HIV+ no MDD (*n* = 5) vs. HIV−/Ctl (*n* = 3)	Frontal cortex	miR and mRNA Microarray	Multiple miRs/multiple neuronal genes
Zhou et al. (2012)	HIV+ with HAD (*n* = 10) vs. HIV+ no HAD (*n* = 8)	Frontal cortex	miR Microarray	Multiple miRs/neuronal axon-guidance genes
Xu et al. (2017)	HIV+ with HAND and HIVE (*n* = 10) vs. HIV+ HAND, no HIVE (*n* = 10) vs. HIV−/Ctl (*n* = 10)	Midfrontal gyrus (cortex)	miRNA microarray	17 miRs/genes of peroxisome biogenesis
Desplats et al. (2014)	HIV+ METH+ (*n* = 13) vs. HIV+ no METH (*n* = 14)	Frontal cortex	DNA methylation array	METH increases global DNA methylation
Levine et al. (2016)	HIV+ with HAND (*n* = 43) vs. HIV+/CN (*n* = 15)	Dorso-lateral, medial frontal, occipital cortex	DNA methylation array	HIV affects epige-netic clock, HAND associated with accelerated aging
Plasma and peripheral blood				
Kadri et al. (2016)	HIV+ with NCI (*n* = 17) vs. HIV+/CN (*n* = 13)	Plasma	miRNA microarray, qRT-PCR	4 miR pairs discern NCI from CN
Asahchop et al. (2016)	Discovery cohort: HIV+ HAND (*n* = 22) vs. HIV+/CN (*n* = 25) Validation cohort: HIV+ HAND (*n* = 12) vs. HIV+/CN (*n* = 12)	Plasma	miRNA microarray, qRT-PCR	miR-3665, miR-4516, miR-4707–5p/neural develop-ment, cell death, cytokine, genes
Shiau et al. (2021)	HIV+ (*n* = 69) vs. HIV−/Ctl (*n* = 38)	Peripheral blood	DNA methylation array	HIV accelerates epigenetic clock, impairs cognition
Total *n* in epigenetic studies	HIV+ with or without NCI or HIVE (*n* = 314) vs. HIV−/Ctl (*n* = 56)			

**TABLE 4:** Genetic Studies. List of studies performed using *post-mortem* brain tissues or blood-derived cells of HIV-infected and uninfected individuals. AAN, American Academy of Neurology, criteria for HAD diagnosis; AD, Alzheimer’s Disease; CN, cognitively normal; COMT, catechol O-methyl transferase; CSA, cognitive score for age; CSF, cerebrospinal fluid; DSM, Diagnostic and statistical manual of mental disorders; DTI, diffusion tensor imaging (neuroimaging); Frascati, Frascati criteria for HAND classification (CN; ANI, asymptomatic neurocognitive impairment; MND, mild cognitive disorder; HAD); GCS, global cognitive score; GDS, global deficit score; HIV^−^/Ctl, uninfected, healthy control; HAE, HIV-1-associated encephalopathy; HAND, HIV-associated neurocognitive disorders; HIVE, HIVE encephalitis; HAND, HIV associated neurocognitive disorders; NCI, Neurocognitive impairment; HAD, HIV-associated dementia; HIV+/CN, HIV+ cognitive normal subjects; LE, leukoencephalopathy; MCMD, minor cognitive and motor disorder; MDD, major depressive disorder; METH, methamphetamine; MRI, magnetic resonance imaging (neuroimaging); MSK, Memorial Sloan Kettering classification of HAD; NP, neuropsychological; NPI-O, neuropsychologically impaired but not definitely caused by HIV infection due to comorbid factor; PBMC, peripheral blood mononuclear cells; qRT-PCR, quantitative reverse transcription polymerase chain reaction, RFLP, restriction fragment length polymorphism; SNV, single-nucleotide variants; WES, whole-exome sequencing; WB, Western blotting; WGA, whole genome amplification.

Study (References)	Groups included in the study (number of cases)	Area(s) of the brain or other tissue	Methods 1) NCI diagnosis 2) genetics	Gene, SNP, SNV, association (y = yes/n = no)
Dunlop et al. (1997)	HIV+ dementia (*n* = 32) or possible dementia (*n* = 24) vs. HIV+ no dementia (*n* = 73); dementia not defined as HAD; Paper mentions total *n* = 132 but data are shown for *n* = 129	frontoparietal cortex, cerebral white matter, hippocampus, cerebellum, basal ganglia, brain stem, thoracic spinal cord	1) HAD graded by physician: no dementia, possible dementia, dementia	ApoEε4 (n) for dementia and HIVE
			2) ApoE PCR and RFLP	
Corder et al. (1998)	HIV+ (*n* = 44) with (*n* = 11) or without dementia (*n* = 32) (neurological data for *n* = 43; dementia not defined as HAD)	Serum	1) NP testing	ApoEε4 (y)
			2) ApoE Western blotting	
Sato-Matsumura et al. (1998)	HIV+ HIVE or LE (*n* = 44) vs. HIV+ no HIVE or LE (*n* = 30)) vs. HIV-/Ctl (*n* = 35)	Brain tissue and PBMC	1) None, HIVE and/or LE	TNFα promotor (n), HLA-DR3 (n)
			2) PCR for SNPs	
Boven et al. (1999)	HIV+ HAD/ADC (*n* = 9) or HIV+ no HAD (*n* = 8) vs. HIV−/Ctl (*n* = 6)	Frontal cortex	1) HAD/ADC: MSK	CCR5 ± Δ32 (y)
			2) RT-PCR for CCR5 ± Δ32	
van Rij et al. (1999)	HIV+ HAD/ADC (*n* = 49) vs. HIV+ no HAD/ADC (*n* = 186) HIV−/Ctl (*n* = 5)	PBMC	1) ADC (yes/no) 2) PCR and RFLP for CCR5 ± Δ32 and CCR2 ± 64I	CCR5 ± Δ32 (y), CCR2 ± 64I (n)
Quasney et al. (2001)	HIV+ with HAD (*n* = 16) vs. HIV+ no HAD (*n* = 45) vs. HIV−/Ctl (*n* = 231)	Brain tissue and PBMC	1) HAND/HAD: AAN, MSK	TNFα promotor (y)
			2) PCR for SNP TNFα promotor	
Gonzalez et al. (2002)	Adults: HIV+ with or without HAD (*n* = 1,151); Children: HIV+ with or without HAD (*n* = 592) vs. HIV−/Ctl (*n* = 270)	PBMC	1) ADC (yes/no) 2) PCR for SNP, RFLP for CCL2	CCL2 (y) depending on genotype/SNP
Singh et al. (2003)	Children: HIV+ with or without neurological deterioration (*n* = 1,049)	PBMC	1) Neurological and neurocognitive decline	CCR5wt/Δ32 (y), CCR5 SNPs (y), CCR2-64I (y), CXCL12 (y)
			2) PCR and RFLP for SNPs	
Cutler et al. (2004)	HIV+ HAD (*n* = 10)	Brain	1) HAD/ADC: MSK	ApoEε4 (n)
			2) PCR for ApoE	
Diaz-Arrastia et al. (2004)	HIV+ with signs of HIVE (*n* = 270)	Brain	1) None, HIVE	ApoEε4 (n), TNFα (n), IL-1B*2 (n), ILIRN*2 (n)
			2) PCR for SNP, RFLP for	
Singh et al. (2004)	HIV+/CN with follow-up testing for development of NCI (*n* = 121)	PBMC	1) NP testing, GDS	CCR5-wt/Δ32 (n), CCR2-64I (y), CCL2/MCP1 (n)
			2) SNPs PCR and melting curve analysis for	
Valcour et al. (2004)	HIV+ HAD (*n* = 182)	Banked specimen	1) HAD: AAN	ApoEε4 (y) for age ≥ 50 years
			2) PCR and RFLP for ApoE	
Shiramizu et al. (2006)	Children: HIV+ with HIVE (*n* = 27)	CSF	1) HAE	CCL2/MCP1 SNP (y)
			2) PCR for SNP, RFLP	
Burt et al. (2008)	HIV+/CN with follow-up testing for development of NCI/HAD (*n* = 1,267) vs. HIV−/Ctl (*n* = 1,132)	Blood	1) HAD (yes/no)	ApoEε4 (n)
			2) TaqMan PCR-based allelic discrimination	
Pomara et al. (2008)	HIV+ no HAD (*n* = 41)	Blood	1) NP testing	ApoEε4 (y)
			2) PCR and RFLP for ApoE	
Pemberton et al. (2008)	HIV+ HAD/ADC (*n* = 56) For meta-analysis data from: Dunlop et al. (1997); Corder et al. (1998); Quasney et al. (2001), Diaz-Arrastia et al. (2004)	PBMC	1) ADC (mild, moderate, severe, or vegetative)	ApoEε4 (n), IL-1α (n), IL-1β (n), IL-12 (n), TNFα (y)
			2) PCR and RFLP; and meta-analysis	
Levine et al. (2009)	HIV+ HAD/ADC (*n* = 26) vs. HIV+/CN (*n* = 117)	Brain tissue and PBMC	1) HAD: AAN	CCL3 (y), CXCL12 (n), CCL2 (n), CCL5 (n)
			2) PCR and TaqMan PCR-based allelic discrimination assays for SNPs	IL-1α (n), IL-10 (n), TNFα (n)
Bousman et al. (2010)	HIV ± METH (*n* = 108) vs. HIV−/Ctl ± METH (*n* = 84)	PBMC	1) Executive funct. deficit score	COMT (y)
			2) Multiplex PCR (SpectroCHIP)	
Joska et al. (2010)	HIV+ with different severity of HAND (*n* = 144), HIV−/Ctl (*n* = 50)	Blood	1) HAND: Frascati	ApoEε4 (n)
			2) PCR and RFLP	
Spector et al. (2010)	HIV+ with and without HCV+ (*n* = 201)	Blood	1) GDS	ApoEε4 (y), CCL2(n), CCL3L1(n), CCR5(n), CCR2(n), CXCL12(n), CX3CR1(n), IL-4(n), MBL2 (y)
			2) PCR and RFLP	
Sun et al. (2010)	HIV+ NCI (*n* = 17) vs. HIV+/CN (*n* = 27) vs. HIV−/Ctl/CN (*n* = 11)	PBMC	1) NP impairment	ApoEε4 (n)
			2) PCR and RFLP	
Andres et al. (2011)	HIV+ with different severity of HAND/HAD/GCS (*n* = 48) vs. HIV−/Ctl (*n* = 39)	Blood	1) GCS and HAD scale	ApoEε4 (y)
(HAD scale Power et al. (1995))			2) PCR for genotype	
Chang et al. (2011)	HIV+ with different severity of HAND/HAD/GCS (*n* = 69) vs. HIV−/Ctl (*n* = 70)	Blood	1) NP testing, domain- and global Z-scores	ApoEε4 (y)
			2) PCR for genotype	
Gupta et al. (2011)	HIV+ METH (*n* = 93) vs. HIV+ no METH (*n* = 84) vs. HIV- METH (*n* = 77) vs. HIV- no METH (*n* = 77), each with or without NCI	PBMC	1) NCI, composite deficit score	DRD3 (y) for HIV+ METH group
			2) Multiplex PCR (SpectroCHIP)	
Singh et al. (2011)	Children: HIV+ with various degrees of CNS disease/NCI (*n* = 572)	PBMC	1) Neurological and neurocognitive decline	HLA Class I alleles (y) HLA-Class II (y)
			2) Multiplex WGA (DNA), Luminex 100 platform	
Bol et al. (2012)	HIV+ HAD (*n* = 72) or HIV+ no HAD (*n* = 241); HAD criteria of DSM, AAN, Frascati	PBMC	1) HAD: DSM, AAN, Frascati	Prep1 (y), CCR5-Δ32 (y), CCR2 (n), CCL2 (n), CCL3 (n), ApoE (n), DYRK1A (n), MOAP1 (n), PDE8A (n), SPOCK3 (n), TNFα (n), UBR7 (n)
			2) SNP analysis, chip and PCR methods	
Schrier et al. (2012)	HIV+ (*n* = 203) vs. HIV−/Ctl (*n* = 198); GDS for all	Whole blood	1) HAND: GDS	HLA Class I alleles (y) HLA-DR*04 (y) ApoEε4 (y)
			2) Genotyping for HLA class I and II, ApoE	
Brown et al. (2012)	HIV+ HAD of various degrees based on MSK (*n* = 262)	Buccal swabs or PBMC	1) HAD: MSK	CCL3L1 (n)
			2) qPCR for copy number	
Levine et al. (2012b)	HIV+ with MCMD or HAD (AAN) or ANI (Frascati) or no HAND/NCI (*n* = 184)	PBMC and/or other tissue	1) NP testing and AAN	COMT (n), BDNF (n), DAT (n)
			2) Genotyping/SNPs	
Soontornniyomkij et al. (2012)	HIV+ with HAND/ANI (Frascati) or MCMD/HAD (AAN) (*n* = 160) vs. HIV−/Ctl (*n* = 22)	Brain tissue	1) HAND/HAD: AAN, Frascati	ApoEε4 (y)
			2) ApoE genotype	
Morgan et al. (2013)	HIV+ with HAND (Frascati) (*n* = 466)	PBMC	1) HAND: Frascati	ApoEε4 (n)
			2) ApoE genotype	
Panos et al. (2013)	HIV+ with MCMD or HAD (AAN) or ANI (Frascati) or no HAND/NCI (*n* = 259)	PBMC and/or other tissue	1) HAND/HAD: AAN, Frascati	ApoEε4 (y) for age ≥ 50 years
			2) ApoE genotype	
Singh et al. (2013)	Children: HIV+ with various degrees of CNS disease/NCI (*n* = 1,049)	PBMC	1) Neurological function	APOBEC3G (y), genotype dependent
			2) APOBEC3G genotype	
Hoare et al. (2013)	HIV+ with ApoEe4 ± vs. HIV+ no ApoEe4 (*n* = 24), neuropsych testing for all	PBMC	1) NP testing	ApoEε4 (y)
			2) ApoE genotype	
Morales et al. (2013)	HIV+ with or without HAND/MCMD/HAD (AAN) (*n* = 20) vs. HIV−/Ctl (*n* = 16)	PBMC(?) and CSF	1) HAND: AAN	YWHAE (y), genotype dependent
			2) Genotype/SNP for YWHAE (qPCR and WB)	
Levine et al. (2014)	HIV+ with or without NCI (*n* = 546) vs. HIV−/Ctl (*n* = 406)	PBMC	1) HAND: Domain T-scores	CCL2 (y), CCL3 (y), CXCL12 (n), MBL (n) HIV+, (y) HIV-/Ctl, COMT (n) HIV+, (y) HIV-/Ctl
			2) Genotyping for SNPs	
Chang et al. (2014)	HIV+ with or without NCI (*n* = 80) vs. HIV−/Ctl (*n* = 97)	Whole blood	1) HAND: NP testing or Frascati	ApoEε4 (y)
			2) ApoE genotype	
Sundermann et al. (2015)	HIV+ with or without NCI (*n* = 54) vs. HIV−/Ctl (*n* = 33), N-back test for memory for all	Not specified	1) N-Back test for memory	COMT (y)
			2) Genotype/SNPs for COMT	
Becker et al. (2015)	HIV+ with or without various degrees of NCI (*n* = 2,846)	PBMC or immortalized B cells	1) HAND: NP testing and T-scores based on Frascati criteria	ApoEε4 (n)
			2) ApoE genotype	
Villalba et al. (2015)	HIV+ with or without various degrees of NCI (*n* = 267), all with history of alcohol use disorder	Whole blood	1) Neurocognitive test battery2) Genotype PCR	DRD2 (y), DRD4 (y)
Total *n* in candidate-gene studies	HIV+ with or without NCI (*n* = 13,296) vs. HIV−/Ctl (*n* = 2,843)			
Levine et al. (2012a)	HIV+ with or without various degrees of NCI/HAND/HAD (AAN and Frascati) (*n* = 1,287)	Not specified	1) Neurocognitive decline: Domain T-scores; HAD: AAN; NCI: Frascati	None
			2) GWAS (∼2.5 million SNPs)	
Jia et al. (2017)	HIV+ with GDS < 0.5 (*n* = 684) or GDS > 0.5 (*n* = 366), with or without various degrees of HAND (Frascati), CN (*n* = 568), ANI (*n* = 359), MND (*n* = 92), HAD (*n* = 30) (total *n* = 1,050)	PBMC	1) HAND: NCI defined by GDS GDS < 0.5 and GDS > 0.5, continuous GDS; Frascati	None for GDS-defined NCI and HAND; SH3RF3 (y) for GDS < 0.5 vs. GDS > 0.5; T-cell receptor α locus (y) for continuous GDS
			2) GWAS (SNPs)	
Hulgan et al. (2019)	HIV+ with GDS < 0.5 (*n* = 658) or GDS > 0.5 (*n* = 352), with or without various degrees of HAND (Frascati), CN (*n* = 553), HAND (*n* = 457) (total *n* = 1,010)	Whole blood	1) HAND: NCI defined by GDS > 0.5, continuous GDS; Frascati	mtDNA copy number (y) with NCI defined by GDS > 0.5, continuous GDS and HAND
			2) GWAS (SNPs) and mtDNA sequencing	
Rawat et al. (2020)	Children: Discovery cohort: HIV+ with NCI; (CSA < 70; *n* = 217) or without NCI (CSA > 70; *n* = 247); Validation cohort 1: HIV+ (*n* = 394) vs. HIV-/Ctl (*n* = 214) Validation cohort 2: HIV+ (*n* = 357)	PBMC	1) CSA2) WES for identification of SNVs associated with NCI	CCRL2 (y); RETREG1/FAM134B (y); YWHAH (y)
Total *n* in GWAS/WES studies	HIV+ with or without NCI (*n* = 4,543) vs. HIV−/Ctl (*n* = 214)			

In this review, we summarize the work that has shaped our current understanding of genetic underpinnings and changes in gene expression in HAND patients and their use for identification of disease mechanism. Additionally, we discuss the use of transcriptomic studies in animal models that can lead to the discovery of genes of interest for disease mechanisms and that can subsequently be validated in humans.

## Transcriptomic Profiles of HIV-Associated Neurocognitive Disorders, HIV Encephalitis, and HIV^+^ Brain Samples

### Transcriptomic Alterations in HIV Encephalitis Patients

There are currently nine published reports determining whole-genome expression changes using human brain samples from HIV^+^ individuals in order to determine changes in protein-coding RNAs, and cellular and molecular pathways altered during HAND or HIV encephalitis (HIVE) (Gelman et al., 2004; Masliah et al., 2004; Shapshak et al., 2004; Everall et al., 2005; Borjabad et al., 2011; Gelman et al., 2012; Zhou et al., 2012; Levine et al., 2013; Canchi et al., 2020). Table 1 summarizes the study groups of interest, areas of the brain, and methods used for each publication. Table 2 summarizes the gene networks and functions that are differentially regulated or otherwise have been linked to NCI, HAD, HAND and/or HIVE. A subset of the studies is briefly discussed below.

Two of the earliest gene expression studies focused on HIV^+^ brains with and without HIVE, which was diagnosed during autopsy based on the presence of multinucleated giant cells, microgliosis, astrocytosis, microglial nodules, and myelin parlor (Masliah et al., 2004; Everall et al., 2005). Using this classification, Masliah et al. (2004) showed 74 genes were significantly downregulated, and 59 were significantly upregulated in association with encephalitis. Most of the genes downregulated were axonal and dendritic components involved in regulating synaptic plasticity, spine formation, and synaptic transmission. While most of the genes upregulated were part of the interferon response. These changes suggested that changes in synapto-dendritic function could be a causative driver of HIVE (Masliah et al., 2004).

Everall et al. (2005) used the same comparison of HIV+ brains with and without HIVE, but added the variable of methamphetamine use, which is a frequent comorbidity found in PLWH (Everall et al., 2005; Sanchez and Kaul, 2017). Similar gene expression changes were observed between the HIV^+^ brains without and those with HIVE as observed previously (Masliah et al., 2004); however, the new transcriptomic analysis revealed an increase in interferon related genes, STAT1, IFITMI, Tyro3, ISG15, and IFP35 in the HIVE + methamphetamine group (Everall et al., 2005).

### Transcriptomic Alterations Associated With Neurocognitive Impairment in People Living with HIV

Although the presence of HIVE is a clearly distinguishable effect of HIV on the brain, many subjects with HAND do not present histopathological features of HIVE (Everall et al., 2009; Gelman et al., 2013). For that matter, while the number of infiltrating macrophages and activated microglia, and loss of synapses and neuronal dendrites correlate positively, neuronal apoptosis, number of infected cells in the brain, and levels of HIV RNA in plasma or cerebrospinal fluid do not significantly correlate or predict neurocognitive impairment (NCI) (Glass et al., 1995; Adle-Biassette et al., 1999; Gelman et al., 2013).

Gelman et al. (2004) was the first gene expression study to use the presence of cognitive deficits to define their study groups. The investigation focused on changes in ion channels and their regulatory proteins. A total of 18 ion channel-related proteins were differentially regulated (six genes upregulated and 12 genes downregulated) (Gelman et al., 2004). Hence, it was proposed that alteration in neuronal firing could lead to neurocognitive changes while not requiring neuronal loss. Moreover, such a scenario could explain why people with HAND do not always develop HIVE or neuronal loss (Adle-Biassette et al., 1999; Gelman et al., 2004). Interestingly, genome-wide analysis of HIVE brains indicated alterations in several ion channel-related proteins (Masliah et al., 2004), but only a calcium channel, voltage-dependent, L-type α 1B subunit was found to be altered in both, the microarrays for HIVE (Masliah et al., 2004) and MCMD/HAD (Gelman et al., 2004). One caveat of these comparisons beyond the difference in classification of study groups is that Masliah et al. (2004) did not use un-infected (HIV^−^) samples as a control (Masliah et al., 2004).

To characterize the differences between HIV^+^/NCI and HIV^+^/NCI with HIVE, in one of the most comprehensive studies to date, Gelman et al. (2012) analyzed samples from the neocortex, neostriatum, and white matter from 1) un-infected individuals (HIV^−^), 2) cognitively normal HIV^+^ (HIV^+^/CN), 3) HIV^+^/NCI without HIVE, 4) HIV^+^/NCI with HIVE patients (Gelman et al., 2012). This study revealed different transcriptomic profiles and altered biological pathways for each group (Table 2). HIVE with NCI was characterized by high viral RNA load in the brain, increased interferon signaling, and activation of interferon regulatory factors (IRF) and cytosolic pattern recognition receptors in all brain regions, accompanied by a decrease in pathways associated with neurotransmission in the neocortex. On the other hand, NCI without HIVE was associated with low viral RNA load in the brain, and with changes of microvascular endothelial and perivascular cells, pointing to neurovascular stress. Interestingly, only weak immune responses and a lack of altered neurotransmission-related gene expression was observed, although slightly altered pathways may have remained undetected due to power limitations. Interestingly, HIV^+^ without HIVE or NCI samples showed alterations in synaptic systems in the neostriatum, which could suggest that adaptation in some neuronal circuitries prevented impairment (Gelman et al., 2012).

Transcriptomic studies have also contributed to our understanding of the interplay between HAND and cART. Borjabad et al. (2011) showed that the primary factor driving the clustering of HAND patients’ transcriptome patterns was whether individuals were on cART at the time of death. Transcriptional changes in the white matter of untreated HAND patients with and without HIVE were similar with inflammatory and immune pathways being up-regulated and neuronal signaling pathways being down-regulated (Borjabad et al., 2011). Consistent with previous studies (Masliah et al., 2004; Everall et al., 2005), HIVE showed greater dysregulation in immune response pathways. Interestingly, the group with HAND but cART had up to 14-fold fewer altered genes, highlighting a striking effect of cART on gene expression in HIV infection and the intensity of the dysregulation. Although, closer to HIV-seronegative samples, cART with HAND groups had 260 genes significantly altered. By comparing overlapping genes between treated and untreated HAND patients, this study was able to discern a pattern associated with neurocognitive impairments independent of viral load. These genes were related to innate and adaptive immune responses, class II MHC, defects in myelin metabolism, and interferon response.

In a separate meta-analysis, (Borjabad and Volsky, 2012) compared their microarray data with those of earlier gene expression studies of HIVE and HAND/HAD and also investigated overlap with differential gene expression in Alzheimer’s Disease (AD) and Multiple Sclerosis (MS) (Borjabad and Volsky (2012). The meta-analysis found overlaps between HAND and each of the other neurodegenerative diseases, and a small group of genes differentially regulated in all three conditions, but HAND and AD shared relatively more changes related to neuronal function whereas HAND and MS overlapped more on regulated genes of immunity (Borjabad and Volsky, 2012) (Table 2).

Zhou et al. (2012) analyzed the frontal cortex of 10 HIV^+^ individuals with HAD and seven without dementia to determine genome-wide changes in mRNA. A total of 468 transcripts were differentially regulated. Database for Annotation, Visualization and Integrated Discovery (DAVID) determined that gene expression changes were associated with various biological functions, such as cell death/cell cycle, neuronal processes, metabolism, and others. Interestingly, 29% of altered genes were related to neuronal components, like axon, neuron projection, and dendrites. Using a different bioinformatic analysis approach (analysis of all transcripts using Gene Set Enrichment Analysis (GSEA) to determine significantly altered GO (gene ontology) gene sets) revealed changes in similar biological functions, such as dendrites, ATPase activity in the transmembrane, intracellular transport, mRNA binding, and others. Zhou et al. (2012), went a step further, and randomly selected five samples of the HAD and HIV^+^ non-dementia groups, and profiled changes in microRNAs (miRs) between the two groups. Analysis of 68 significantly altered miRNA and their target mRNAs using DAVID showed similar pathways as those observed using the mRNA data. Altogether, miRNA analysis revealed the dysregulation of microtubule assembly, ion channels, in particular Ca^2+^, Na^+^, and glutamine transport, and others. Joint analysis of mRNA and miRNA changes revealed alterations in axon guidance and the downstream signaling of MAPK and Ca^2+^ signaling pathways (Zhou et al., 2012).

Additionally, Levine et al. (2013), used 21 of the 24 samples of the study by Gelman et al. (2012). The key difference was the use of weighted gene co-expression network analysis (WGCNA) to determine the relationship between gene expression changes and NCI regardless of HIVE and CNS penetration effectiveness (CPE) score of cART, and the investigation in a meta-analysis of overlap of differential gene expression between HAND and AD. The results not only validated the original observations of Gelman et al. (2012), but also identified several genes that correlated with NCI and CPE, and networks associated with oligodendrocyte and mitochondria functioning as significantly altered (Levine et al., 2013). Moreover, the meta-analysis found overlap between HAND and AD in the dysregulation of gene networks, including those for mitochondria.

It remains important to better understand the factors driving the differences between asymptomatic neurocognitive impairment (ANI), mild neurocognitive disorder (MND), and HIV-associated dementia (HAD). Canchi et al., 2020 investigated the differences in gene expression between MND and cognitive normal HIV^+^ individuals using RNA-seq to study human brain tissue from HAND patients (Canchi et al., 2020). RNA-seq offers several advantages including the ability to assay non-coding RNAs (miRNA, lncRNA, etc.) in addition to mRNA, while at the same time overcoming some of the limitations of microarrays (background noise and saturation associated with hybridization, incorrect annotation of probes, poor isoform coverage, etc.). Employing RNA-seq, the study identified over 1,000 genes altered in HIV^+^ brains with MND. Top upregulated genes were related to immune response and viral replication, while the most down regulated genes were involved with synaptic maintenance. Interestingly, disease-gene associations implicated AD and tauopathy, suggesting possible shared mechanisms between the two pathologies. Additionally, transcription factor analysis found c/EBPβ and most of its targets to be altered in MND and determined that a number of these changes were astrocyte-specific (Canchi et al., 2020).

### General Considerations Regarding the Transcriptomic Studies of HIV-Associated Neurocognitive Disorders and HIV Encephalitis

While the here reviewed studies reported numerous differentially regulated genes and gene networks in association with HAND and/or HIVE and also overlap with AD and MS, the conclusions are based on the analysis of a total of 143 cases of HIV infection with or without NCI or HIVE and 29 HIV^−^ controls, and some of the studies appear to have shared study subjects, e.g., (Borjabad et al., 2011; Gelman et al., 2012; Levine et al., 2013). In comparison to transcriptomic studies of AD and MS analyzed by Borjabad et al. (Borjabad and Volsky, 2012), the studies for HAND and HIVE seem extremely underpowered. Moreover, only five of the studies included uninfected controls (Gelman et al., 2004; Shapshak et al., 2004; Borjabad et al., 2011; Gelman et al., 2012; Levine et al., 2013). The omission of uninfected controls seems problematic, because an HIV^+^ individual that tested neurocognitively normal *pre-mortem* may have deteriorated between the last neurocognitive assessment and the time of death, or could already have alterations in gene expression associated with imminent, as of yet undetectable NCI. Altogether, the interpretation of the transcriptomic studies is associated with a lot of uncertainty for three major reasons: 1) the variety of criteria and approaches employed for the assessment of HIV-associated NCI and HAND; 2) the overall limited number of cases that were comprehensively characterized for neurocognitive performance and for which corresponding high-quality CNS RNA is available and, 3) the omission of HIV-negative control subjects in many investigations. Thus overall, it seems desirable to analyze an increasing number of samples from neurocognitively impaired PWLH and uninfected controls by microarray or RNA-seq. Revealing gene-expression dependent neural mechanisms underlying NCI and HAND will require the *post-mortem* analysis of brain tissues. While those samples may be available in sufficient numbers from the pre-cART era, the fact that cART has decreased morbidity and mortality associated with HIV infection and thus fewer cases come to autopsy, may pose a challenge to obtaining a sufficient number of specimen from age-matched, cART-treated HIV^+^ individuals (Palella et al., 1998; McArthur et al., 2010; Heaton et al., 2011; Vella et al., 2012; Saylor et al., 2016).

## Epigenetic Studies of HIV-Associated Neurocognitive Disorders

Epigenetics describes by definition heritable changes of gene expression that are not associated with alterations of the regulated DNA sequence itself. While DNA methylation and histone modifications are two well established mechanisms mediating epigenetic modifications, non-coding microRNAs (miRs) seem to be associated with epigenetics in two ways. First, miRs affect epigenetic regulation and, second miRs are subject to it (Chuang and Jones, 2007). We found 14 studies specifically addressing epigenetics in the context of HAND, 10 analyzing miRs, three DNA methylation and one a histone deacetylase (HDAC). Four of the studies used an *in vitro* model, three plasma or peripheral blood and six *post-mortem* brain tissue. Table 3 summarizes the epigenetic studies, but given the prominence of miR studies we discuss only those in more detail below.

### Micro RNAs

Non-coding RNA dysregulation can contribute to the development of neurocognitive diseases, in particular, miRs influence transcription by stabilizing mRNA and preventing translation (Juzwik et al., 2019). Genome-wide transcriptomic techniques, such as microarrays and RNA-seq, allow investigating changes in miRs and other non-coding RNAs; however, only a few studies have used human brain samples to study alterations in miRs during HAND.

Two of the *in vitro* studies used neuronal cell cultures exposed to HIV proteins and analyzed alterations of miRNA profiles (Eletto et al., 2008; Mukerjee et al., 2011). Eletto et al. found differential regulation of miR-128a, which affects the synaptic protein SNAP25 (Eletto et al., 2008) while Mukerjee et al. observed differential regulation of multiple miRs that are predicted to affect multiple neuronal genes (Mukerjee et al., 2011). A third study by Dave and Khalili (2010) investigated the effect of morphine treatment on miRs in human monocyte-derived macrophages and deduced the effect these changes may have on HIV neuropathogenesis. The study identified 26 miRs, with hsa-miR15b and miR181b as most up- and down-regulated, respectively, by morphine. Follow-up experiments investigated potential miR targets and found a decrease of fibroblast growth factor (FGF)-2 and an increase in CCL8 and IL-6 induced by morphine, linking the drug to inflammation with the potential to contribute to peripheral cell infiltration, inflammation, and thus, to worsening AIDS dementia (Dave and Khalili, 2010).

Yelamanchili et al. (2010) identified miR-21, miR-142-3p, and miR142-5p as significantly dysregulated in the caudate nucleus (striatum) in samples of patients with HIVE and HAD, and hippocampus and striatum of SIV-infected Rhesus monkeys (Yelamanchili et al., 2010). The study also showed that miR-21 expression increases in neurons of SIV-infected rhesus monkeys. Stimulation of NMDA receptors *in vitro* led to an increased expression of miR-21. In turn, miR-21 expression increased outward K^+^ current *in vitro* and downregulated myocyte enhancer factor 2C (MEF2C), a crucial factor in neuronal function and survival, both *in vitro* and *in vivo* (Yelamanchili et al., 2010).

The study by Tatro et al. also identified multiple miRs differentially regulated by HIV infection that are predicted to modulate neuronal genes, but focused very narrowly on major depressive disorder (MDD) rather than HAND (Tatro et al., 2010).

Noorbakhsh et al. (2010) analyzed the frontal lobe region of HIVE patients and compared it to age- and sex-matched non-infected controls. To determine the functional significance of the altered miRNA, the Sanger miRNA database prediction algorithm was used to predict the mRNA affected by the significantly dysregulated miRNA. The predicted mRNA affected were in turn used to predict biological pathways and functions altered in HIVE brains. Up-regulated miRNAs were mainly associated with genes linked to immune response and inflammation, as well as nucleotide metabolism, and cell cycle pathways. Paradoxically, many downregulated miRNAs were also linked to inflammation (Noorbakhsh et al., 2010). This speaks to the fact that several factors canonically associated with inflammation such as IFNβ, CXCL12, CCL5, CCL4, and others can play neuroprotective roles in HAND (Kaul and Lipton, 1999; Kaul et al., 2007; Medders et al., 2010; Sanchez et al., 2016b; Thaney et al., 2017; Ojeda-Juárez et al., 2020). Interestingly, cell death-related genes were the second-largest type of genes affected by downregulated miRNAs. Caspases were associated with multiple miRNAs decreased in HIVE brains, and consistent with the microarray analysis, caspase 6 was verified to be significantly upregulated in HIVE brains (Noorbakhsh et al., 2010).

The study by Zhou et al. (2012) combined analysis of mRNA and miRs and was discussed above under transcriptomic analyses (Zhou et al., 2012).

Xu et al. (2017) identified 17 significantly altered miRNA in neocortical brain tissues of HAND patients (the group included an equal number of HIVE and non-HIVE samples) and compared it to samples from HIV^+^ individuals without HAND. Of the miRNA altered, miR-500a-5p, miR-34c-3p, miR-93-3p, and miR-381-3p were predicted to target genes involved in peroxisome biogenesis. These miRNAs were also increased in primary macrophages following HIV infection. Their expression led to a decrease of peroxisomal proteins, alteration in morphology of the peroxisomes, and increased expression of immune genes, such as IFIT2, IFI16, viperin, IRF1, and OAS1 (Xu et al., 2017). Interestingly, even when Xu et al., (2017) included HIVE samples in the analysis, there was no overlap with miRNA observed in Noorbakhsh et al. (2010). An important caveat is the fact that Xu et al. (2017) included HAND patients without HIVE but not un-infected samples as control Xu et al., (2017). Not surprisingly, this suggests that there are changes in brain miRNA during HIV infection.

MicroRNAs have also been investigated as biomarkers for HAND. Plasma samples from two cohorts of HIV^+^ patients with and without HAND were used to identify differentially regulated miRNAs using a microarray-based analysis. MicroRNA-3665, miR-4516, and miR-4707–5p were altered in both cohorts. Interestingly, miR-3665, and miR-4516 were able to predict HAND, and appeared to be better predictors than CD4^+^ T-cell count, viral load, and central nervous system (CNS) penetration effectiveness (CPE) score of cART (Asahchop et al., 2016). Kadri et al. identified four microRNA pairs that best distinguished NCI from non-NCI groups (miR-495-3p/let-7b-5p, miR-495-3p/miR-151-5p, miR-495-3p/miR-744-5p and miR-376a-3p/miR-16-5p) (Kadri et al., 2016). Interestingly, the authors found no association between the expression of these microRNA-pairs and any of the clinical and demographic variables of the study subjects. However, two sets of microR-pairs provided better sensitivity and specificity for discrimination of NCI from non-NCI groups: miR-376a-3p/miR-532-3p combined with either miR-495-3p/miR-744-5p or miR-495-3p/let-7b-5p.

### General Considerations Regarding the Epigenetic Studies of HIV-Associated Neurocognitive Disorders

The 10 epigenetic studies using human tissue samples, involved a total of 314 HIV^+^ cases with or without NCI or HIVE and 56 uninfected controls (Table 3). While this is more cases than the mRNA-based transcriptomic studies discussed above, it will most likely require a much larger number of cases to confirm the miRs and DNA methylation patterns that have so far been implicated in HIV-associated NCI and HAND. Thus, interpretation of the epigenetic studies is affected by the same limitations as the transcriptomic data.

## Genetic Candidate-Gene and Genome-wide Association Studies of HIV-Associated Neurocognitive Disorders

In order to understand the host genetics of HIV-1 disease several groups have undertaken a number of genome-wide association studies (GWAS) which have identified numerous genetic variants in parts of the immune system that affect susceptibility to HIV-1 infection and the course of HIV-1 disease, such as parts of the histocompatibility complex and factors influencing the ratio of CD4^+^ to CD8^+^ T-cells (Ferreira et al., 2010; van Manen et al., 2012; McLaren et al., 2013; McLaren et al., 2015; Gingras et al., 2020). It is certainly reasonable to argue that all those factors are likely to influence the development of HAND. We have identified 40 studies that focused on the association of a limited number of candidate-genes with HIV-related NCI and HAND but only four GWAS. All those genetic studies are summarized in Table 4. Unfortunately, the four GWAS aimed specifically at HAND have so far been inconclusive using the past and current criteria for HAND diagnosis and a limited number of fully sequenced samples for individuals that were also comprehensively characterized for neurocognitive performance (Levine et al., 2012a; Jia et al., 2017; Hulgan et al., 2019; Rawat et al., 2020). However, the GWAS have provided some support for previously studied candidate genes and suggested some new ones by analyzing the genes that came closest to the strict statistically determined cut-offs for significance in GWAS and by using GDS rather than HAND criteria for grouping cases with NCI (Jia et al., 2017; Hulgan et al., 2019). Below we will first discuss the most frequently studied candidate genes and then the GWAS summarized in Table 4.

### Candidate-Gene Studies for Neurocognitive Effects of HIV and HIV-Associated Neurocognitive Disorders

The candidate gene studies for HAND have most prominently investigated single nucleotide polymorphisms (SNP) or other variations in genes of the immune system (e.g., CCR5, CCR2, CCL2, CCL3, CCL5, members of HLA complex, TNFα), lipid metabolism (ApoEε4) or the dopaminergic system (e.g., DAT, DRD2, DRD3, COMT, BDNF). Table 4 also indicates whether or not a study found an association with any aspect of HAND (indicated as (y) or (n)) because different investigations frequently came to different conclusions for the same candidate gene.

The most studied candidate gene variant to date is ApoEε4, a gene important in lipid metabolism and a widely accepted risk factor for AD (Jansen et al., 2019). ApoEε4 was investigated by 20 genetic studies for HAND, of which 10 concluded it had a significant effect on NCI. Interestingly, five of the studies concluded that the effect was age-dependent in that a detrimental effect of ApoEε4 on neurocognition was found for HIV-infected individuals of 50 years and older (Valcour et al., 2004; Chang et al., 2011; Soontornniyomkij et al., 2012; Panos et al., 2013; Chang et al., 2014). Another aspect that may have influenced the outcomes of the investigations is that of the 10 studies finding an association of ApoEε4 with HAND, five included uninfected controls, and of the 10 reports concluding no effect of the gene only two involved HIV^−^ subjects. ApoEε4 seems of particular interest as it addresses the question of whether or not HAND shares pathological mechanisms with AD or, if HIV-infection can facilitate the development of AD.

The CCR5Δ32 deletion or a SNP in the HIV co-receptor was investigated in six studies, of which four concluded a significant effect on HIV associated NCI in that the CCR5Δ32 deletion provided protection against deterioration of neurocognition, HAD or HAND (Boven et al., 1999; van Rij et al., 1999; Singh et al., 2003; Bol et al., 2012). Of the four studies that found an association of CCR5 with NCI, only one included uninfected controls. Neither of the two reports that found no effect of CCR5 of the gene on NCI involved HIV^−^ subjects.

CCR2 genotypes were analyzed in five studies, none of which included uninfected controls. However, two investigations reported an effect of CCR2 genotype on HIV-associated NCI (Singh et al., 2003; Singh et al., 2004).

Genotypes of the ligand for CCR2, CCL2, were part of seven studies, of which three found an effect on HAND/NCI/HAD, and two of the three investigations included HIV^−^ control subjects (Gonzalez et al., 2002; Shiramizu et al., 2006; Levine et al., 2014). In contrast, none of the four analyses which concluded that CCL2 genotypes lack an influence on HAND involved HIV^−^ subjects.

The genotype of one of the natural ligands of CCR5, CCL3, was found to affect neurocognitive performance in two out of three studies (Levine et al., 2009; Levine et al., 2014). One of the two studies observing an effect included uninfected controls.

Genetic variants of TNFα were examined in six studies, with two concluding an effect on the severity of HAD based on criteria pre-dating the Frascati classification of HAND (Quasney et al., 2001; Pemberton et al., 2008). One of the studies included HIV^−^ controls (Quasney et al., 2001), while the second included the first as part of a meta-analysis for comparison to results of newly analyzed cases (Pemberton et al., 2008). None of the other analyses of TNFα genotypes included uninfected controls.

Genetic variants of a number of dopaminergic genes were explored in six studies for playing a role in HIV-associated NCI. Four studies detected effects of genotypes in the context of HIV and neurocognition, two studies for COMT (Bousman et al., 2010; Sundermann et al., 2015), one for DRD3 (Gupta et al., 2011) and one for DRD2 and -4 (Villalba et al., 2015). Three of the four studies included uninfected individuals. Of the two analyses that did not find an association between dopaminergic genotypes and HIV-associated alterations of neurocognition, one noticed that dopamine-related gene variants only affected HIV^−^ control subjects (Levine et al., 2014).

### Genome-wide Association Studies of HIV-Associated Neurocognitive Disorders

A first GWAS based on 1,287 participants of the Multicenter AIDS Cohort Study (MACS) found no genetic susceptibility loci associated with deteriorating neurocognition, such as decline in executive functioning or speed of processing, when analyzing 2.5 million SNPs, and no association between SNPs and NCI or HAD (Levine et al., 2012a). Another GWAS for HAND including 1,050 participants of the Anti-Retroviral Therapy Effects Research (CHARTER) study found no SNPs with genome-wide significance for NCI and HAND defined by a global deficit score (GDS) (Jia et al., 2017). Classification of samples using a binary GDS (*n* = 366 GDS ≥ 0.5 and *n* = 684 GDS < 0.5) identified two SNPs in chromosome 2 for the gene *SH3RF3*, which has been implicated in cortical thickness and surface measures based on neuroimaging (www.genecards.org). In addition, two SNPs were detected for FBN3 and CCL25, and four SNPs, including the most significant one, were found in chromosome 14 in the locus for the α-chain of the T-cell receptor. A third GWAS involving 1,010 participants of the CHARTER study investigated the association of mitochondrial DNA (mtDNA) copy numbers in blood with HAND, since this parameter has been linked to neurocognitive function in individuals without HIV-1 infection (Hulgan et al., 2019). MtDNA copy number was deduced from genome-wide genotyping as the ratio of mtDNA probe intensities relative to nuclear DNA SNP signal in peripheral blood cells. Lower mtDNA copy number was associated with longer cART, but not exposure to dideoxynucleoside anti-retroviral drugs. Overall mtDNA copy number associated with GDS-defined NCI and HAND. In all analyses, higher mtDNA copy number in peripheral blood was associated with poorer cognitive performance, suggesting that increased mitochondrial replication may be due to mitochondrial dysfunction (Hulgan et al., 2019). A fourth GWAS investigated subjects of two pediatric HIV cohorts using a cognitive score for age (CSA) assessment and whole-exome sequencing (WES) to identify single nucleotide variants (SNVs) associated with NCI (Rawat et al., 2020). This study associated SNVs in three genes, CCRL2, RETREG1/FAM134B and YWHAH, with the risk of NCI and the level of inflammation.

### General Considerations Regarding the Genetic Studies of HIV-Associated Neurocognitive Disorders

Similarly to the transcriptomic and epigenetic studies discussed above, the interpretation of the genetic studies, both of candidate genes and GWAS, are hampered by three major issues: 1) the variety of criteria and approaches employed for the assessment of HIV-associated NCI and HAND; 2) the overall limited cases that were comprehensively characterized for neurocognitive performance and for which genomic DNA is available or has been fully sequenced and, 3) the omission of HIV-negative control subjects in many investigations. The candidate-gene studies included in aggregate 13,296 HIV^+^ subjects with or without NCI, but only 2,843 HIV^−^ controls. The GWAS examined 4,543 HIV^+^ subjects with or without NCI, and 214 HIV^−^ controls. For comparison, a recent GWAS meta-analysis of genetic risk factors for AD included 21,982 confirmed disease cases and 41,944 cognitively normal controls derived from 63 studies (Kunkle et al., 2019).

## Meta-Analysis of Transcriptomic Studies

One of the advantages of genome-wide expression analysis is the requirement by most scientific journals and major research funding agencies to make the data sets publicly available. This allows for the re-assessment of data as analytical techniques improve, the combination of multiple studies to increase statistical power, and the comparison between different diseases. Borjabad and Volsky (2012) employed the combined data from most transcriptomic studies of human brain samples of HIV^+^ individuals up to the time point of the study (Masliah et al., 2004; Shapshak et al., 2004; Everall et al., 2005; Borjabad et al., 2011) and determined overlap in gene expression with Alzheimer’s disease (AD), and multiple sclerosis (MS). The results showed that there was a 35% overlap in genes dysregulated between AD and HAND samples, and a 13% overlap between MS and HAND. Surprisingly, the most significant biological pathways shared between HIV and AD were immune response, defense response (up-regulated), and transmission of nerve impulse, and synaptic impulse (down-regulated). Alterations in neuronal communication are believed to underlie both AD and HAND, even when HAND is virally induced, and AD is hypothesized to be driven by protein misfolding and aggregation. Adding to the evidence of pathways shared between AD and HAND, (Levine et al., 2013), reanalyzed the data published by Gelman et al. (2012) to determine shared transcriptional patterns between the two diseases. Genes altered in both were related to mitochondrial function and cancer, and they were involved in several pathways including down-regulation of energy pathways, mitochondria, transit peptide, and synaptic vesicle, as well as upregulation in cell differentiation, cell communication, regulation of transcription, and phosphorylation (Levine et al., 2013).

Another meta-analysis was conducted with the data published by Gelman et al. (2012) exploring the differences in gene expression between groups that were not analyzed in the original work. Sanna et al. (2017) identified pathways associated with tissue damage, RNA transcription and processing, and pathways associated with protein degradation to be altered in HIV^+^ cases with NCI but without HIVE when compared to HIV^+^ cognitively normal samples (Sanna et al., 2017).

## Cross-Validation of Human Whole-Genome Transcriptomics With Animal Models

Animal models are essential to study human diseases. They allow to better control for variables and test detailed hypotheses that would be impossible to address with humans in real life settings. Also, an *in vivo* model can lead to the discovery of novel genes or mechanisms that can be later validated in humans. It is imperative that these animal models are validated by comparison to disease relevant human samples. One of the methods of validation is the comparison of transcriptomic changes in humans versus animal models. For example, Maung et al. (2014) used a genome-wide CNS expression analysis of a transgenic mouse model expressing the HIV gp120 protein under the GFAP promoter in the brain (Toggas et al., 1994; Maung et al., 2014) and compared the differential gene expression pattern to those reported by Gelman et al. (2012). There was a significant overlap between the differentially expressed genes in the HIVgp120-transgenic mouse and the human HIV^+^ brains. The mouse microarray led to the identification of lipocalin-2 (Lcn2) as a gene of interest (Maung et al., 2014). Subsequently, Lcn2 expression was shown to correlate with neuropathology and compromised neurocognitive performance in a subset of HIV^+^ individuals, and with decreased cortical thickness and area (Williams et al., 2019; Ojeda-Juárez et al., 2020; Williams et al., 2020). The studies demonstrated in *in vivo* and *in vitro* model systems that elevated levels of LCN2 exert neurotoxicity contributing to HIV brain injury (Ojeda-Juárez et al., 2020; Williams et al., 2020). The microarray analysis of the HIV gp120-transgenic brains found several additional genes and their associated biological pathways that were also differentially regulated in their HIV^+^ human counterparts. These pathways included the interferon response, activation of NFkB by virus, CCR5 in macrophages, and GABA receptors, among others (Maung et al., 2014). Follow up studies analyzing targeted groups of genes with quantitative reverse transcription polymerase chain reaction (qRT-PCR) arrays also verified significant changes in expression of genes related to neurotransmission (Ojeda-Juárez et al., 2020; Singh et al., 2020).

Additionally, transcriptomic studies in other animal models, such as the simian immunodeficiency virus (SIV) infected monkeys, have identified pathways similarly altered in humans, providing another valuable model for discovery of disease mechanisms at the molecular level (Winkler et al., 2012). A study of gene expression changes at 2 weeks post inoculation showed an increase in cytokine signaling, and interferon related pathways (Roberts et al., 2004). Recently, RNA-seq revealed that four poly (ADP-ribose) polymerases (PARPs) were upregulated in the frontal cortex of Rhesus macaques with detectable SIV, suggesting a role in the pathways of neuroinflammation (Mavian et al., 2021).

Moreover, the above discussed study by Yelamanchili et al. (2010) provides a similar example of the use of animal models for epigenetics. That investigation identified miR-21, miR-142-3p, and miR142-5p as significantly dysregulated in the caudate nucleus (striatum) in samples of patients with HIVE and HAD, and hippocampus and striatum of SIV-infected Rhesus monkeys (Yelamanchili et al., 2010).

## Conclusion

The interpretation of transcriptomic, epigenetic and genetic studies of HAND are currently limited by three major issues: 1) the variety of criteria and approaches employed for the assessment of HIV-associated NCI and HAND; 2) the overall limited number of cases that were comprehensively characterized for neurocognitive performance and for which high quality RNA or genomic DNA is available, or has been fully sequenced and, 3) the omission of HIV-negative control subjects in many investigations.

The here reviewed studies indicate a clear need for better overall national and international standardization of neurocognitive assessments. Various modifications have been proposed for a better consensus on the diagnosis of neurocognitive impairment in PLWH, such as an adjustment of the limit score used to distinguish ANI from normal performance, treating cognitive performance as a continuous variable, and including clinical history in the diagnosis (Saloner and Cysique, 2017; Ciccarelli, 2020; Nightingale et al., 2021). While it may seem a monumental task, those proposals could be implemented retroactively for cases where the raw data is available and that can be re-analyzed. A recent GWAS study has started to implement some of those proposed modifications (Hulgan et al., 2019). An alternative approach to circumnavigate the ANI controversy could be to focus transcriptomic, epigenetic and genetic studies of HAND on the most agreed-on categories of neurocognitive impairment and only include MND and HAD cases. Once patterns of gene expression, and epigenetic and genetic features have been established using those cases, ANI samples could be re-evaluated in order to discern those that share a part of or approach properties of clearly established MND and HAD.

Genome-wide gene expression studies have been a useful tool for identification of the wide-ranging effects of HIV-1 infection on the brain. The differences between histopathological features, such as encephalitis, and HAND remain to be characterized by transcriptomics, epigenetics and genetics. Accumulating transcriptomic data can eventually provide the basis for meta-analyses that can lead to identification of shared mechanisms across neurodegenerative diseases, and to cross-validate animal models which are important to the study of disease mechanisms.

The adoption of next generation sequencing techniques is still not widespread for the study of HAND, and surprisingly only one study, (Canchi et al., 2020), has used RNA-seq, and one by Rawat et al. (2020) which employed WES, to study HIV-associated NCI/HAND. The RNA-seq study also inferred astrocyte-specific changes by determining that differentially regulated c/EBPβ targets astrocytic marker genes (Canchi et al., 2020). With the combined RNA-seq and bioinformatics approach the study overcame one of the limitations of using homogenized brain tissue, which is the inability to discern cell type-specific changes and contributions to pathological mechanisms. One possible solution and future improvement is to use single cell RNA-seq to determine changes in different cell types. However, banked tissues pose some technical challenges to single cell RNA-seq in terms of isolating cell types to homogeneity, and RNA integrity. However, samples may be available in sufficient numbers from the pre-cART era. In contrast, the fact that cART has decreased morbidity and mortality associated with HIV infection with fewer cases coming to autopsy may pose a challenge to obtain a sufficient number of specimen from age-matched, cART-treated HIV^+^ individuals. Studying virally controlled, cART treated individuals is essential to better understand the current status of HIV disease and HAND, but it remains also important to continue studying non-treated cases since access to antiretroviral therapy is not universal and in order to make comparisons with other neurodegenerative diseases for which there is currently no effective therapy, such as AD. Future experiments will also need to address the differences in multiple brain regions. Most studies to date have been examining frontal cortex, and only two studies have used more than one region (Gelman et al., 2004; Gelman et al., 2012). This can prevent the discovery of region-specific changes leading to the development of HAND.

The number and availability of samples from individuals that have been characterized for neurocognition in a detailed and standardized manner seems to pose another limitation for the wide use of functional genomics in the study of HAND. It is crucial to remember that even in well controlled studies, a small sample size can obfuscate results. On this subject, one must commend the valuable resources provided by the National NeuroAIDS Tissue Consortium (NNTC). The NNTC recruits HIV^+^ and HIV^−^ individuals as future tissue donors, collects psychological and psychiatric data and biofluids *ante-mortem,* and collects, characterizes, and stores *post mortem* specimen for research (Morgello et al., 2001; Cserhati et al., 2015). It is perhaps a reflection of the history of the HIV epidemic that participant recruitment focused on very sick individuals, including many with numerous comorbidities, while comparable numbers of controls were harder to come by and therefore lacking. Nevertheless, the NNTC made it possible to ask targeted questions based on neuropathological features or level of cognitive decline. However, there is still a need to increase the number of samples to obtain sufficient power to answer the question of genetic underpinnings and pathological mechanisms responsible for the development of HAND. In contrast to the transcriptomic profiling, genetic studies do not require brain tissues, and the combined 17,839 HIV^+^ cases examined in previous candidate-gene studies and GWAS may provide a sufficient basis to move forward (even if there was some overlap between samples analyzed in the two approaches). Nineteen GWAS studies analyzing 41,912 subjects have been performed for HIV infection and gene associations with viral load setpoint, HIV susceptibility, replication, disease progression, and AIDS but without consideration of HAND/HAD/NCI (Gingras et al., 2020). Yet these GWAS may provide a framework in which genetic data for cases that have been neurocognitively and/or neuropathologically assessed could be placed, even if the neurocognitive assessment occurred in a variety of ways. We already know that neurocognitive impairment and neuropathology do not necessarily coincide. But it will be interesting to see with which of the genetic patterns linked to divergent susceptibility to HIV disease the neurocognitively and neuropathologically characterized cases match, if any, or if they form their own pattern(s).

## References

[B1] Adle-Biassette H. Chrétien F. Wingertsmann L. Héry C. Ereau T. Scaravilli F. (1999). Neuronal Apoptosis Does Not Correlate With Dementia in HIV Infection but Is Related to Microglial Activation and Axonal Damage. Neuropathol. Appl. Neurobiol. 25, 123–133. 10.1046/j.1365-2990.1999.00167.x 10216000

[B2] Andres M. A. Feger U. Nath A. Munsaka S. Jiang C. S. Chang L. (2011). APOE ε4 Allele and CSF APOE on Cognition in HIV-Infected Subjects. J. Neuroimmune Pharmacol. 6, 389–398. 10.1007/s11481-010-9254-3 21184197 PMC4899041

[B3] Antinori A. Arendt G. Becker J. T. Brew B. J. Byrd D. A. Cherner M. (2007). Updated Research Nosology for HIV-Associated Neurocognitive Disorders. Neurology. 69, 1789–1799. 10.1212/01.wnl.0000287431.88658.8b 17914061 PMC4472366

[B4] Asahchop E. L. Akinwumi S. M. Branton W. G. Fujiwara E. Gill M. J. Power C. (2016). Plasma microRNA Profiling Predicts HIV-Associated Neurocognitive Disorder. AIDS. 30, 2021–2031. 10.1097/qad.0000000000001160 27191977

[B5] Barre-Sinoussi F. Chermann J. Rey F. Nugeyre M. Chamaret S. Gruest J. (1983). Isolation of a T-Lymphotropic Retrovirus From a Patient at Risk for Acquired Immune Deficiency Syndrome (AIDS). Science. 220, 868–871. 10.1126/science.6189183 6189183

[B6] Becker J. T. Kingsley L. Mullen J. Cohen B. Martin E. Miller E. N. (2009). Vascular Risk Factors, HIV Serostatus, and Cognitive Dysfunction in Gay and Bisexual Men. Neurology. 73, 1292–1299. 10.1212/wnl.0b013e3181bd10e7 19841381 PMC2764414

[B7] Becker J. T. Martinson J. J. Penugonda S. Kingsley L. Molsberry S. Reynolds S. (2015). No Association Between Apoε4 Alleles, HIV Infection, Age, Neuropsychological Outcome, or Death. J. Neurovirol. 21, 24–31. 10.1007/s13365-014-0290-2 25388225 PMC4319994

[B8] Bol S. M. Booiman T. van Manen D. Bunnik E. M. van Sighem A. I. Sieberer M. (2012). Single Nucleotide Polymorphism in Gene Encoding Transcription Factor Prep1 Is Associated With HIV-1-Associated Dementia. PLoS One. 7, e30990. 10.1371/journal.pone.0030990 22347417 PMC3274517

[B9] Borjabad A. Morgello S. Chao W. Kim S.-Y. Brooks A. I. Murray J. (2011). Significant Effects of Antiretroviral Therapy on Global Gene Expression in Brain Tissues of Patients With HIV-1-Associated Neurocognitive Disorders. Plos Pathog. 7, e1002213. 10.1371/journal.ppat.1002213 21909266 PMC3164642

[B10] Borjabad A. Volsky D. J. (2012). Common Transcriptional Signatures in Brain Tissue From Patients With HIV-Associated Neurocognitive Disorders, Alzheimer's Disease, and Multiple Sclerosis. J. Neuroimmune Pharmacol. 7, 914–926. 10.1007/s11481-012-9409-5 23065460 PMC3515772

[B11] Bousman C. A. Cherner M. Atkinson J. H. Heaton R. K. Grant I. Everall I. P. (2010). COMT Val158Met Polymorphism, Executive Dysfunction, and Sexual Risk Behavior in the Context of HIV Infection and Methamphetamine Dependence. Interdiscip. Perspect. Infect. Dis. 2010, 678648. 10.1155/2010/678648 20069120 PMC2804107

[B12] Boven L. A. Bruggen T. Sweder van Asbeck B. Marx J. J. M. Nottet H. S. L. M. (1999). Potential Role of CCR5 Polymorphism in the Development of AIDS Dementia Complex. FEMS Immunol. Med. Microbiol. 26, 243–247. 10.1111/j.1574-695x.1999.tb01395.x 10575135

[B13] Brown A. Skolasky N. Sacktor K. Marder K. Cohen G. Schifitto R. L. (2012). CCL3L1 Gene Copy Number in Individuals With and Without HIV-Associated Neurocognitive Disorder. Curr. Biomark Find. 2012, 1–6. 10.2147/cbf.s27685 23814703 PMC3693394

[B14] Burt T. D. Agan B. K. Marconi V. C. He W. Kulkarni H. Mold J. E. (2008). Apolipoprotein (Apo) E4 Enhances HIV-1 Cell Entry *In Vitro*, and the APOE 4/4 Genotype Accelerates HIV Disease Progression. Proc. Natl. Acad. Sci. 105, 8718–8723. 10.1073/pnas.0803526105 18562290 PMC2438419

[B15] Canchi S. Swinton M. K. Rissman R. A. Fields J. A. (2020). Transcriptomic Analysis of Brain Tissues Identifies a Role for CCAAT Enhancer Binding Protein β in HIV-Associated Neurocognitive Disorder. J. Neuroinflammation. 17, 112. 10.1186/s12974-020-01781-w 32276639 PMC7149918

[B16] Chang L. Andres M. Sadino J. Jiang C. S. Nakama H. Miller E. (2011). Impact of Apolipoprotein E ε4 and HIV on Cognition and Brain Atrophy: Antagonistic Pleiotropy and Premature Brain Aging. Neuroimage. 58, 1017–1027. 10.1016/j.neuroimage.2011.07.010 21803164 PMC3171637

[B17] Chang L. Jiang C. Cunningham E. Buchthal S. Douet V. Andres M. (2014). Effects of APOE 4, Age, and HIV on Glial Metabolites and Cognitive Deficits. Neurology. 82, 2213–2222. 10.1212/wnl.0000000000000526 24850492 PMC4113464

[B18] Chuang J. C. Jones P. A. (2007). Epigenetics and microRNAs. Pediatr. Res. 61, 24R–29R. 10.1203/pdr.0b013e3180457684 17413852

[B19] Ciccarelli N. (2020). Considerations on Nosology for HIV-Associated Neurocognitive Disorders: it Is Time to Update? Infection. 48, 37–42. 10.1007/s15010-019-01373-8 31691905

[B20] Ciccarelli N. Fabbiani M. Grima P. Falasca K. Tana M. Baldonero E. (2013). Comparison of Cognitive Performance in HIV or HCV Mono-Infected and HIV-HCV Co-Infected Patients. Infection. 41, 1103–1109. 10.1007/s15010-013-0503-2 23839213

[B21] Corder E. H. Robertson K. Lannfelt L. Bogdanovic N. Eggertsen G. Wilkins J. (1998). HIV-Infected Subjects With the E4 Allele for APOE Have Excess Dementia and Peripheral Neuropathy. Nat. Med. 4, 1182–1184. 10.1038/2677 9771753

[B22] Crystal H. Kleyman I. Anastos K. Lazar J. Cohen M. Liu C. (2012). Effects of Hepatitis C and HIV on Cognition in Women. J. Acquir Immune Defic Syndr. 59, 149–154. 10.1097/qai.0b013e318240566b 22107817 PMC3319079

[B23] Cserhati M. F. Pandey S. Beaudoin J. J. Baccaglini L. Guda C. Fox H. S. (2015). The National NeuroAIDS Tissue Consortium (NNTC) Database: an Integrated Database for HIV-Related Studies. Database (Oxford)., 1–10. 10.1093/database/bav074 PMC4520230 26228431

[B24] Cutler R. G. Haughey N. J. Tammara A. McArthur J. C. Nath A. Reid R. (2004). Dysregulation of Sphingolipid and Sterol Metabolism by ApoE4 in HIV Dementia. Neurology. 63, 626–630. 10.1212/01.wnl.0000134662.19883.06 15326233

[B25] Dave R. S. Khalili K. (2010). Morphine Treatment of Human Monocyte-Derived Macrophages Induces Differential miRNA and Protein Expression: Impact on Inflammation and Oxidative Stress in the Central Nervous System. J. Cell. Biochem. 110, 834–845. 10.1002/jcb.22592 20564181 PMC2923828

[B26] Desplats P. Dumaop W. Cronin P. Gianella S. Woods S. Letendre S. (2014). Epigenetic Alterations in the Brain Associated With HIV-1 Infection and Methamphetamine Dependence. PLoS One. 9, e102555. 10.1371/journal.pone.0102555 25054922 PMC4108358

[B27] DeVaughn S. Müller-Oehring E. M. Markey B. Brontë-Stewart H. M. Schulte T. (2015). Aging With HIV-1 Infection: Motor Functions, Cognition, and Attention - A Comparison with Parkinson's Disease. Neuropsychol. Rev. 25, 424–438. 10.1007/s11065-015-9305-x 26577508 PMC5519342

[B28] Diaz-Arrastia R. Gong Y. Kelly C. J. Gelman B. B. (2004). Host Genetic Polymorphisms in Human Immunodeficiency Virus-Related Neurologic Disease. J. Neurovirol. 10 Suppl 1 (Suppl. 1), 67–73. 10.1080/753312755 14982742

[B29] Dunlop O. Goplen A. K. Liestol K. Myrvang B. Rootwelt H. Christophersen B. (1997). HIV Dementia and Apolipoprotein E. Acta Neurol. Scand. 95, 315–318. 10.1111/j.1600-0404.1997.tb00217.x 9188909

[B30] Eletto D. Russo G. Passiatore G. Del Valle L. Giordano A. Khalili K. (2008). Inhibition of SNAP25 Expression by HIV‐1 Tat Involves the Activity of Mir‐128a. J. Cell. Physiol. 216, 764–770. 10.1002/jcp.21452 18381601 PMC2662126

[B31] Everall I. Salaria S. Roberts E. Corbeil J. Sasik R. Fox H. (2005). Methamphetamine Stimulates Interferon Inducible Genes in HIV Infected Brain. J. Neuroimmunology. 170, 158–171. 10.1016/j.jneuroim.2005.09.009 16249037

[B32] Everall I. Vaida F. Khanlou N. Lazzaretto D. Achim C. Letendre S. (2009). Cliniconeuropathologic Correlates of Human Immunodeficiency Virus in the Era of Antiretroviral Therapy. J. Neurovirol. 15, 360–370. 10.3109/13550280903131915 20175693 PMC3078805

[B33] Ferreira M. A. R. Mangino M. Brumme C. J. Zhao Z. Z. Medland S. E. Wright M. J. (2010). Quantitative Trait Loci for CD4:CD8 Lymphocyte Ratio Are Associated With Risk of Type 1 Diabetes and HIV-1 Immune Control. Am. J. Hum. Genet. 86, 88–92. 10.1016/j.ajhg.2009.12.008 20045101 PMC2801744

[B34] Foley J. Ettenhofer M. Wright M. J. Siddiqi I. Choi M. Thames A. D. (2010). Neurocognitive Functioning in HIV-1 Infection: Effects of Cerebrovascular Risk Factors and Age. The Clin. Neuropsychologist. 24, 265–285. 10.1080/13854040903482830 PMC2863992 20162495

[B35] Force G. Ghout I. Ropers J. Carcelain G. Marigot-Outtandy D. Hahn V. (2021). Improvement of HIV-Associated Neurocognitive Disorders After Antiretroviral Therapy Intensification: the Neuro+3 Study. J. Antimicrob. Chemother. 76, 743–752. 10.1093/jac/dkaa473 33179033

[B36] Gallo R. Salahuddin S. Popovic M. Shearer G. Kaplan M. Haynes B. (1984). Frequent Detection and Isolation of Cytopathic Retroviruses (HTLV-III) From Patients With AIDS and at Risk for AIDS. Science. 224, 500–503. 10.1126/science.6200936 6200936

[B37] Gelman B. B. Chen T. Lisinicchia J. G. Soukup V. M. Carmical J. R. Starkey J. M. (2012). The National NeuroAIDS Tissue Consortium Brain Gene Array: Two Types of HIV-Associated Neurocognitive Impairment. PLoS One. 7, e46178. 10.1371/journal.pone.0046178 23049970 PMC3458860

[B38] Gelman B. B. Lisinicchia J. G. Morgello S. Masliah E. Commins D. Achim C. L. (2013). Neurovirological Correlation with HIV-Associated Neurocognitive Disorders and Encephalitis in a HAART-Era Cohort. J. Acquir Immune Defic Syndr. 62, 487–495. 10.1097/qai.0b013e31827f1bdb 23242157 PMC3664102

[B39] Gelman B. B. Soukup V. M. Schuenke K. W. Keherly M. J. Holzer C. 3rd Richey F. J. (2004). Acquired Neuronal Channelopathies in HIV-Associated Dementia. J. Neuroimmunology. 157, 111–119. 10.1016/j.jneuroim.2004.08.044 15579287

[B40] Gingras S. N. Tang D. Tuff J. McLaren P. J. (2020). Minding the gap in HIV Host Genetics: Opportunities and Challenges. Hum. Genet. 139, 865–875. 10.1007/s00439-020-02177-9 32409920 PMC7272494

[B41] Glass J. D. Fedor H. Wesselingh S. L. McArthur J. C. (1995). Immunocytochemical Quantitation of Human Immunodeficiency Virus in the Brain: Correlations With Dementia. Ann. Neurol. 38, 755–762. 10.1002/ana.410380510 7486867

[B42] Global HIV and AIDS statistics (2020). The Joint United Nations Programme on HIV/AIDS (UNAIDS), Global HIV & AIDS Statistics — 2020 Fact Sheet.

[B43] Gonzalez E. Rovin B. H. Sen L. Cooke G. Dhanda R. Mummidi S. (2002). HIV-1 Infection and AIDS Dementia Are Influenced by a Mutant MCP-1 Allele Linked to Increased Monocyte Infiltration of Tissues and MCP-1 Levels. Proc. Natl. Acad. Sci. 99, 13795–13800. 10.1073/pnas.202357499 12374865 PMC129777

[B44] Grant I. Franklin D. R. Jr. Deutsch R. Woods S. P. Vaida F. Ellis R. J. (2014). Asymptomatic HIV-Associated Neurocognitive Impairment Increases Risk for Symptomatic Decline. Neurology. 82, 2055–2062. 10.1212/wnl.0000000000000492 24814848 PMC4118496

[B45] Gupta S. Bousman C. A. Chana G. Cherner M. Heaton R. K. Deutsch R. (2011). Dopamine Receptor D3 Genetic Polymorphism (rs6280TC) Is Associated With Rates of Cognitive Impairment in Methamphetamine-Dependent Men With HIV: Preliminary Findings. J. Neurovirol. 17, 239–247. 10.1007/s13365-011-0028-3 21491142 PMC3151555

[B46] Hahn B. H. Shaw G. M. Arya S. K. Popovic M. Gallo R. C. Wong-Staal F. (1984). Molecular Cloning and Characterization of the HTLV-III Virus Associated With AIDS. Nature. 312, 166–169. 10.1038/312166a0 6095086

[B47] Heaton R. K. Franklin D. R. Franklin D. R. Ellis R. J. McCutchan J. A. Letendre S. L. (2011). HIV-Associated Neurocognitive Disorders Before and During the Era of Combination Antiretroviral Therapy: Differences in Rates, Nature, and Predictors. J. Neurovirol. 17, 3–16. 10.1007/s13365-010-0006-1 21174240 PMC3032197

[B48] Hoare J. Westgarth-Taylor J. Fouche J.-P. Combrinck M. Spottiswoode B. Stein D. J. (2013). Relationship between Apolipoprotein E4 Genotype and white Matter Integrity in HIV-Positive Young Adults in South Africa. Eur. Arch. Psychiatry Clin. Neurosci. 263, 189–195. 10.1007/s00406-012-0341-8 22825739

[B49] Hulgan T. Kallianpur A. R. Guo Y. Barnholtz-Sloan J. S. Gittleman H. Brown T. T. (2019). Peripheral Blood Mitochondrial DNA Copy Number Obtained From Genome-Wide Genotype Data Is Associated With Neurocognitive Impairment in Persons With Chronic HIV Infection. J. Acquir Immune Defic Syndr. 80, e95–e102. 10.1097/qai.0000000000001930 30531306 PMC6391216

[B50] Jansen I. E. Savage J. E. Watanabe K. Bryois J. Williams D. M. Steinberg S. (2019). Genome-Wide Meta-Analysis Identifies New Loci and Functional Pathways Influencing Alzheimer's Disease Risk. Nat. Genet. 51, 404–413. 10.1038/s41588-018-0311-9 30617256 PMC6836675

[B51] Jia P. Zhao Z. Hulgan T. Bush W. S. Samuels D. C. Bloss C. S. (2017). Genome-Wide Association Study of HIV-Associated Neurocognitive Disorder (HAND): A CHARTER Group Study. Am. J. Med. Genet. 174, 413–426. 10.1002/ajmg.b.32530 28447399 PMC5435520

[B52] Joseph J. A. Colosi D. R. Rao V. (2016). HIV-1 Induced CNS Dysfunction: Current Overview and Research Priorities. Curr. HIV Res. 14, 389–399. 10.2174/1570162x14666160324124940 27009096

[B53] Joska J. A. Combrinck M. Valcour V. G. Hoare J. Leisegang F. Mahne A. C. (2010). Association Between Apolipoprotein E4 Genotype and Human Immunodeficiency Virus-Associated Dementia in Younger Adults Starting Antiretroviral Therapy in South Africa. J. Neurovirol. 16, 377–383. 10.3109/13550284.2010.513365 20825268

[B54] Juzwik C. A. S S. D. Zhang Y. Paradis-Isler N. Sylvester A. Amar-Zifkin A. (2019). microRNA Dysregulation in Neurodegenerative Diseases: A Systematic Review. Prog. Neurobiol. 182, 101664. 10.1016/j.pneurobio.2019.101664 31356849

[B55] Kadri F. LaPlante A. De Luca M. Doyle L. Velasco-Gonzalez C. Patterson J. R. (2016). Defining Plasma MicroRNAs Associated With Cognitive Impairment in HIV-Infected Patients. J. Cell. Physiol 231, 829–836. 10.1002/jcp.25131 26284581 PMC4758906

[B56] Kaul M. Lipton S. A. (1999). Chemokines and Activated Macrophages in HIV Gp120-Induced Neuronal Apoptosis. Proc. Natl. Acad. Sci. 96, 8212–8216. 10.1073/pnas.96.14.8212 10393974 PMC22214

[B57] Kaul M. Ma Q. Medders K. E. Desai M. K. Lipton S. A. (2007). HIV-1 Coreceptors CCR5 and CXCR4 Both Mediate Neuronal Cell Death but CCR5 Paradoxically Can Also Contribute to protection. Cell Death Differ. 14, 296–305. 10.1038/sj.cdd.4402006 16841089

[B58] Kunkle B. W. Grenier-Boley B. Grenier-Boley B. Sims R. Bis J. C. Damotte V. (2019). Genetic Meta-Analysis of Diagnosed Alzheimer's Disease Identifies New Risk Loci and Implicates Aβ, Tau, Immunity and Lipid Processing. Nat. Genet. 51, 414–430. 10.1038/s41588-019-0358-2 30820047 PMC6463297

[B59] Levine A. J. Miller J. A. Shapshak P. Gelman B. Singer E. J. Hinkin C. H. (2013). Systems Analysis of Human Brain Gene Expression: Mechanisms for HIV-Associated Neurocognitive Impairment and Common Pathways With Alzheimer's Disease. BMC Med. Genomics. 6, 4. 10.1186/1755-8794-6-4 23406646 PMC3626801

[B60] Levine A. J. Quach A. Moore D. J. Achim C. L. Soontornniyomkij V. Masliah E. (2016). Accelerated Epigenetic Aging in Brain Is Associated With Pre-Mortem HIV-Associated Neurocognitive Disorders. J. Neurovirol. 22, 366–375. 10.1007/s13365-015-0406-3 26689571 PMC4900944

[B61] Levine A. J. Reynolds S. Reynolds S. Cox C. Miller E. N. Sinsheimer J. S. (2014). The Longitudinal and Interactive Effects of HIV Status, Stimulant Use, and Host Genotype upon Neurocognitive Functioning. J. Neurovirol. 20, 243–257. 10.1007/s13365-014-0241-y 24737013 PMC4040160

[B62] Levine A. J. Service S. Miller E. N. Reynolds S. M. Singer E. J. Shapshak P. (2012a). Genome-Wide Association Study of Neurocognitive Impairment and Dementia in HIV-Infected Adults. Am. J. Med. Genet. 159B, 669–683. 10.1002/ajmg.b.32071 22628157 PMC3418456

[B63] Levine A. J. Sinsheimer J. S. Bilder R. Shapshak P. Singer E. J. (2012b). Functional Polymorphisms in Dopamine-Related Genes: Effect on Neurocognitive Functioning in HIV+ Adults. J. Clin. Exp. Neuropsychol. 34, 78–91. 10.1080/13803395.2011.623118 22082040 PMC4361028

[B64] Levine A. Singer E. J. Sinsheimer J. S. Hinkin C. H. Papp J. Dandekar S. (2009). CCL3 Genotype and Current Depression Increase Risk of HIV-Associated Dementia. Neurobehavioral HIV Med. Vol. 1, 1–7. 10.2147/nbhiv.s6820 PMC2923399 20725607

[B65] listed N. a. (1991). Nomenclature and Research Case Definitions for Neurologic Manifestations of Human Immunodeficiency Virus-Type 1 (HIV-1) Infection. Neurology. 41, 778. 10.1212/wnl.41.6.778 2046917

[B66] Loewenstein R. J. Sharfstein S. S. (1983). Neuropsychiatric Aspects of Acquired Immune Deficiency Syndrome. Int. J. Psychiatry Med. 13, 255–260. 10.2190/repy-d2wd-07nm-mbrt 6671858

[B67] Masliah E. Roberts E. S. Langford D. Everall I. Crews L. Adame A. (2004). Patterns of Gene Dysregulation in the Frontal Cortex of Patients With HIV Encephalitis. J. Neuroimmunology. 157, 163–175. 10.1016/j.jneuroim.2004.08.026 15579294

[B68] Maung R. Hoefer M. M. Sanchez A. B. Sejbuk N. E. Medders K. E. Desai M. K. (2014). CCR5 Knockout Prevents Neuronal Injury and Behavioral Impairment Induced in a Transgenic Mouse Model by a CXCR4-Using HIV-1 Glycoprotein 120. J. Immunol. 193, 1895–1910. 10.4049/jimmunol.1302915 25031461 PMC4370188

[B69] Mavian C. Ramirez-Mata A. S. Dollar J. J. Nolan D. J. Cash M. White K. (2021). Brain Tissue Transcriptomic Analysis of SIV-Infected Macaques Identifies Several Altered Metabolic Pathways Linked to Neuropathogenesis and Poly (ADP-Ribose) Polymerases (PARPs) as Potential Therapeutic Targets. J. Neurovirol. 27, 101–115. 10.1007/s13365-020-00927-z 33405206 PMC7786889

[B70] McArthur J. C. Steiner J. Sacktor N. Nath A. (2010). Human Immunodeficiency Virus-Associated Neurocognitive Disorders: Mind the gap. Ann. Neurol. 67, 699–714. 10.1002/ana.22053 20517932

[B71] McLaren P. J. Coulonges C. Bartha I. Lenz T. L. Deutsch A. J. Bashirova A. (2015). Polymorphisms of Large Effect Explain the Majority of the Host Genetic Contribution to Variation of HIV-1 Virus Load. Proc. Natl. Acad. Sci. USA. 112, 14658–14663. 10.1073/pnas.1514867112 26553974 PMC4664299

[B72] McLaren P. J. Coulonges C. Ripke S. van den Berg L. Buchbinder S. Carrington M. (2013). Association Study of Common Genetic Variants and HIV-1 Acquisition in 6,300 Infected Cases and 7,200 Controls. Plos Pathog. 9, e1003515. 10.1371/journal.ppat.1003515 23935489 PMC3723635

[B73] Medders K. E. Sejbuk N. E. Maung R. Desai M. K. Kaul M. (2010). Activation of P38 MAPK Is Required in Monocytic and Neuronal Cells for HIV Glycoprotein 120-Induced Neurotoxicity. J. Immunol. 185, 4883–4895. 10.4049/jimmunol.0902535 20855878 PMC3848867

[B74] Morales D. Hechavarria R. Wojna V. Acevedo S. F. (2013). YWHAE/14-3-3ε: a Potential Novel Genetic Risk Factor and CSF Biomarker for HIV Neurocognitive Impairment. J. Neurovirol. 19, 471–478. 10.1007/s13365-013-0200-z 23982958 PMC3962987

[B75] Morgan E. E. Woods S. P. Woods S. P. Letendre S. L. Franklin D. R. Bloss C. (2013). Apolipoprotein E4 Genotype Does Not Increase Risk of HIV-Associated Neurocognitive Disorders. J. Neurovirol. 19, 150–156. 10.1007/s13365-013-0152-3 23408335 PMC3668779

[B76] Morgello S. Gelman B. B. Kozlowski P. B. Vinters H. V. Masliah E. Cornford M. (2001). The National NeuroAIDS Tissue Consortium:a New Paradigm in Brain Banking With an Emphasis on Infectious Disease. Neuropathol. Appl. Neurobiol. 27, 326–335. 10.1046/j.0305-1846.2001.00334.x 11532163

[B77] Mukerjee R. Chang J. R. Del Valle L. Bagashev A. Gayed M. M. Lyde R. B. (2011). Deregulation of microRNAs by HIV-1 Vpr Protein Leads to the Development of Neurocognitive Disorders. J. Biol. Chem. 286, 34976–34985. 10.1074/jbc.m111.241547 21816823 PMC3186354

[B78] Navia B. A. Cho E.-S. Petito C. K. Price R. W. (1986). The AIDS Dementia Complex: II. Neuropathology. Ann. Neurol. 19, 525–535. 10.1002/ana.410190603 3014994

[B79] Navia B. A. Jordan B. D. Price R. W. (1986). The AIDS Dementia Complex: I. Clinical Features. Ann. Neurol. 19, 517–524. 10.1002/ana.410190602 3729308

[B80] Nightingale S. Dreyer A. J. Saylor D. Gisslen M. Winston A. Joska J. A. (2021). Moving on from HAND: Why We Need New Criteria for Cognitive Impairment in People With HIV and a Proposed Way Forward. Clin. Infect. Dis. 73, 1113–1118. 10.1093/cid/ciab366 33904889

[B81] Noorbakhsh F. Ramachandran R. Barsby N. Ellestad K. K. LeBlanc A. Dickie P. (2010). MicroRNA Profiling Reveals New Aspects of HIV Neurodegeneration: Caspase‐6 Regulates Astrocyte Survival. FASEB j. 24, 1799–1812. 10.1096/fj.09-147819 20097875

[B82] Ojeda-Juárez D. Shah R. Fields J. A. Harahap-Carrillo I. S. Koury J. Maung R. (2020). Lipocalin-2 Mediates HIV-1 Induced Neuronal Injury and Behavioral Deficits by Overriding CCR5-Dependent protection. Brain Behav. Immun. 89, 184–199. 10.1016/j.bbi.2020.06.016 32534984 PMC8153086

[B83] Palella F. J. Jr. Delaney K. M. Moorman A. C. Loveless M. O. Fuhrer J. Satten G. A. (1998). Declining Morbidity and Mortality Among Patients With Advanced Human Immunodeficiency Virus Infection. N. Engl. J. Med. 338, 853–860. 10.1056/nejm199803263381301 9516219

[B84] Panos S. Hinkin C. H. Singer E. Thames A. Patel S. M. Sinsheimer J. S. (2013). Apolipoprotein-E Genotype and Human Immunodeficiency Virus-Associated Neurocognitive Disorder: the Modulating Effects of Older Age and Disease Severity. Neurobehavioral HIV Med. 5, 11–22. 10.2147/nbhiv.s39573 PMC4659385 26617462

[B85] Pemberton L. Stone E. Price P. van Bockxmeer F. Brew B. (2008). The Relationship BetweenApoE, TNFA, IL1a, IL1bandIL12bgenes and HIV-1-Associated Dementia. HIV Med. 9, 677–680. 10.1111/j.1468-1293.2008.00614.x 18631256

[B86] Pomara N. Belzer K. D. Silva R. Cooper T. B. Sidtis J. J. (2008). The Apolipoprotein E ɛ4 Allele and Memory Performance in HIV-1 Seropositive Subjects: Differences at Baseline but Not after Acute Oral Lorazepam challenge. Psychopharmacology. 201, 125–135. 10.1007/s00213-008-1253-1 18668226

[B87] Power C. Selnes O. A. Grim J. A. McArthur J. C. (1995). HIV Dementia Scale: a Rapid Screening Test. J. Acquired Immune Deficiency Syndromes Hum. Retrovirology. 8, 273–278. 10.1097/00042560-199503010-00008 7859139

[B88] Price R. W. Brew B. J. (1988). The AIDS Dementia Complex. J. Infect. Dis. 158, 1079–1083. 10.1093/infdis/158.5.1079 3053922

[B89] Quasney M. W. Zhang Q. Sargent S. Mynatt M. Glass J. McArthur J. (2001). Increased Frequency of the Tumor Necrosis Factor-?-308 a Allele in Adults With Human Immunodeficiency Virus Dementia. Ann. Neurol. 50, 157–162. 10.1002/ana.1284 11506397

[B90] Rawat P. Brummel S. S. Singh K. K. Kim J. Frazer K. A. Nichols S. (2020). H.I.V.A.C.S. Pediatric, and A.C.T.N. The International Maternal Pediatric Adolescent, Genomics Links Inflammation With Neurocognitive Impairment in Children Living With Human Immunodeficiency Virus Type-1. J. Infect. Dis. 224, 870–880. 10.1093/infdis/jiaa792 PMC8408770 33373444

[B91] Roberts E. S. Burudi E. M. E. Flynn C. Madden L. J. Roinick K. L. Watry D. D. (2004). Acute SIV Infection of the Brain Leads to Upregulation of IL6 and Interferon-Regulated Genes: Expression Patterns Throughout Disease Progression and Impact on NeuroAIDS. J. Neuroimmunology. 157, 81–92. 10.1016/j.jneuroim.2004.08.030 15579284

[B92] Saiyed Z. M. Gandhi N. Agudelo M. Napuri J. Samikkannu T. Reddy P. V. B. (2011). HIV-1 Tat Upregulates Expression of Histone Deacetylase-2 (HDAC2) in Human Neurons: Implication for HIV-Associated Neurocognitive Disorder (HAND). Neurochem. Int. 58, 656–664. 10.1016/j.neuint.2011.02.004 21315782 PMC3085929

[B93] Saloner R. Cysique L. A. (2017). HIV-Associated Neurocognitive Disorders: A Global Perspective. J. Int. Neuropsychol. Soc. 23, 860–869. 10.1017/s1355617717001102 29198283 PMC5939823

[B94] Saloner R. Heaton R. K. Campbell L. M. Chen A. Franklin D. Jr. Ellis R. J. (2019). Effects of Comorbidity Burden and Age on Brain Integrity in HIV. AIDS. 33, 1175–1185. 10.1097/qad.0000000000002192 30870195 PMC6613389

[B95] Sanchez A. B. Varano G. P. de Rozieres C. M. Maung R. Catalan I. C. Dowling C. C. (2016a). Antiretrovirals, Methamphetamine, and HIV-1 Envelope Protein Gp120 Compromise Neuronal Energy Homeostasis in Association With Various Degrees of Synaptic and Neuritic Damage. Antimicrob. Agents Chemother. 60, 168–179. 10.1128/aac.01632-15 26482305 PMC4704202

[B96] Sanchez A. B. Medders K. E. Maung R. Sánchez-Pavón P. Ojeda-Juárez D. Kaul M. (2016b). CXCL12-Induced Neurotoxicity Critically Depends on NMDA Receptor-Gated and L-Type Ca2+ Channels Upstream of P38 MAPK. J. Neuroinflammation. 13, 252–312. 10.1186/s12974-016-0724-2 27664068 PMC5035480

[B97] Sanchez A. Kaul M. (2017). Neuronal Stress and Injury Caused by HIV-1, cART and Drug Abuse: Converging Contributions to HAND. Brain Sci. 7, 25. 10.3390/brainsci7030025 PMC5366824 28241493

[B98] Sanna P. P. Repunte-Canonigo V. Masliah E. Lefebvre C. (2017). Gene Expression Patterns Associated With Neurological Disease in Human HIV Infection. PLoS One. 12, e0175316. 10.1371/journal.pone.0175316 28445538 PMC5405951

[B99] Sato-Matsumura K. C. Berger J. Hainfellner J. A. Mazal P. Budka H. (1998). Development of HIV Encephalitis in AIDS and TNF-α Regulatory Elements. J. Neuroimmunology. 91, 89–92. 10.1016/s0165-5728(98)00161-1 9846823

[B100] Saylor D. Dickens A. M. Sacktor N. Haughey N. Slusher B. Pletnikov M. (2016). HIV-Associated Neurocognitive Disorder - Pathogenesis and Prospects for Treatment. Nat. Rev. Neurol. 12, 234–248. 10.1038/nrneurol.2016.27 26965674 PMC4937456

[B101] Schrier R. D. Gupta S. Riggs P. Cysique L. A. Letendre S. Jin H. (2012). The Influence of HLA on HIV-Associated Neurocognitive Impairment in Anhui, China. PLoS One. 7, e32303. 10.1371/journal.pone.0032303 22693541 PMC3365058

[B102] Shapshak S. Duncan R. Torres-Munoz J. Duran E. Minagar A. Petito C. (2004). Analytic Approaches to Differential Gene Expression in Aids versus Control Brains. Front. Biosci. 9, 2935–2946. 10.2741/1449 15353327

[B103] Shiau S. Arpadi S. M. Shen Y. Cantos A. Ramon C. V. Shah J. (2021). Epigenetic Aging Biomarkers Associated With Cognitive Impairment in Older African American Adults with HIV. Clin. Infect. Dis. [Epub ahead of print]. 10.1093/cid/ciab563 PMC8664485 34143869

[B104] Shiramizu B. Lau E. Tamamoto A. Uniatowski J. Troelstrup D. (2006). Feasibility Assessment of Cerebrospinal Fluid from HIV-1-Infected Children for HIV Proviral DNA and Monocyte Chemoattractant Protein 1 Alleles. J. Invest. Med. 54, 468–472. 10.2310/6650.2006.06007 17169271

[B105] Singh H. Ojeda-Juárez D. Maung R. Shah R. Roberts A. J. Kaul M. (2020). A Pivotal Role for Interferon-α Receptor-1 in Neuronal Injury Induced by HIV-1. J. Neuroinflammation. 17, 226. 10.1186/s12974-020-01894-2 32727588 PMC7388458

[B106] Singh K. K. Barroga C. F. Hughes M. D. Chen J. Raskino C. McKinney R. E. (2003). Genetic Influence ofCCR5, CCR2,and SDF1Variants on Human Immunodeficiency Virus 1 (HIV‐1)-Related Disease Progression and Neurological Impairment, in Children With Symptomatic HIV‐1 Infection. J. Infect. Dis. 188, 1461–1472. 10.1086/379038 14624371

[B107] Singh K. K. Ellis R. J. Marquie-Beck J. Letendre S. Heaton R. K. Grant I. (2004). CCR2 Polymorphisms Affect Neuropsychological Impairment in HIV-1-Infected Adults. J. Neuroimmunology. 157, 185–192. 10.1016/j.jneuroim.2004.08.027 15579296

[B108] Singh K. K. Gray P. K. Wang Y. Fenton T. Trout R. N. Spector S. A. (2011). HLA Alleles Are Associated With Altered Risk for Disease Progression and central Nervous System Impairment of HIV-Infected Children. J. Acquir Immune Defic Syndr. 57, 32–39. 10.1097/qai.0b013e3182119244 21283014 PMC3107908

[B109] Singh K. K. Wang Y. Gray K. P. Farhad M. Brummel S. Fenton T. (2013). Genetic Variants in the Host Restriction Factor APOBEC3G Are Associated With HIV-1-Related Disease Progression and Central Nervous System Impairment in Children. J. Acquir Immune Defic Syndr. 62, 197–203. 10.1097/qai.0b013e31827ab612 23138837 PMC3658306

[B110] Soontornniyomkij V. Moore D. J. Gouaux B. Soontornniyomkij B. Tatro E. T. Umlauf A. (2012). Cerebral β-amyloid Deposition Predicts HIV-Associated Neurocognitive Disorders in APOE ε4 Carriers. AIDS. 26, 2327–2335. 10.1097/qad.0b013e32835a117c 23018443 PMC3576852

[B111] Spector S. A. Singh K. K. Gupta S. Cysique L. A. Jin H. Letendre S. (2010). APOE ε4 and MBL-2 O/O Genotypes Are Associated With Neurocognitive Impairment in HIV-Infected Plasma Donors. AIDS. 24, 1471–1479. 10.1097/qad.0b013e328339e25c 20442634 PMC3063510

[B112] Sun B. Abadjian L. Rempel H. Calosing C. Rothlind J. Pulliam L. (2010). Peripheral Biomarkers Do Not Correlate With Cognitive Impairment in Highly Active Antiretroviral Therapy-Treated Subjects With Human Immunodeficiency Virus Type 1 Infection. J. Neurovirol. 16, 115–124. 10.3109/13550280903559789 20307252

[B113] Sundermann E. E. Bishop J. R. Rubin L. H. Little D. M. Meyer V. J. Martin E. (2015). Genetic Predictor of Working Memory and Prefrontal Function in Women With HIV. J. Neurovirol. 21, 81–91. 10.1007/s13365-014-0305-z 25515329 PMC4319991

[B114] Tatro E. T. Scott E. R. Nguyen T. B. Salaria S. Banerjee S. Moore D. J. (2010). Evidence for Alteration of Gene Regulatory Networks Through MicroRNAs of the HIV-Infected Brain: Novel Analysis of Retrospective Cases. PLoS One. 5, e10337. 10.1371/journal.pone.0010337 20436668 PMC2859933

[B115] Thaney V. E. O’Neill A. M. Hoefer M. M. Maung R. Sanchez A. B. Kaul M. (2017). IFNβ Protects Neurons From Damage in a Murine Model of HIV-1 Associated Brain Injury. Sci. Rep. 7, 46514. 10.1038/srep46514 28425451 PMC5397848

[B116] Toggas S. M. Masliah E. Rockenstein E. M. Rail G. F. Abraham C. R. Mucke L. (1994). Central Nervous System Damage Produced by Expression of the HIV-1 Coat Protein Gpl20 in Transgenic Mice. Nature. 367, 188–193. 10.1038/367188a0 8114918

[B117] Tross S. Price R. W. Navia B. Thaler H. T. Gold J. Hirsch D. A. (1988). Neuropsychological Characterization of the AIDS Dementia Complex. AIDS. 2, 81–88. 10.1097/00002030-198804000-00002 3132951

[B118] Valcour V. Shikuma C. Shiramizu B. Watters M. Poff P. Selnes O. A. (2004). Age, Apolipoprotein E4, and the Risk of HIV Dementia: the Hawaii Aging With HIV Cohort. J. Neuroimmunology. 157, 197–202. 10.1016/j.jneuroim.2004.08.029 15579298

[B119] van Manen D. van ‘t Wout A. B. Schuitemaker H. (2012). Genome-Wide Association Studies on HIV Susceptibility, Pathogenesis and Pharmacogenomics. Retrovirology. 9, 70. 10.1186/1742-4690-9-70 22920050 PMC3468375

[B120] van Rij R. P. Portegies P. Hallaby T. Lange J. M. Visser J. de Roda Husman A. M. (1999). Reduced Prevalence of the CCR5 Delta32 Heterozygous Genotype in Human Immunodeficiency Virus-Infected Individuals With AIDS Dementia Complex. J. Infect. Dis. 180, 854–857. 10.1086/314940 10438379

[B121] Vella S. Schwartländer B. Sow S. P. Eholie S. P. Murphy R. L. (2012). The History of Antiretroviral Therapy and of its Implementation in Resource-Limited Areas of the World. AIDS. 26, 1231–1241. 10.1097/qad.0b013e32835521a3 22706009

[B122] Villalba K. Devieux J. G. Rosenberg R. Cadet J. L. (2015). DRD2 and DRD4 Genes Related to Cognitive Deficits in HIV-Infected Adults Who Abuse Alcohol. Behav. Brain Funct. 11, 25. 10.1186/s12993-015-0072-x 26307064 PMC4549947

[B123] Wang Y. Liu M. Lu Q. Farrell M. Lappin J. M. Shi J. (2020). Global Prevalence and burden of HIV-Associated Neurocognitive Disorder. Neurology. 95, e2610–e2621. 10.1212/wnl.0000000000010752 32887786

[B124] Williams M. E. Ipser J. C. Stein D. J. Joska J. A. Naudé P. J. W. (2019). The Association of Immune Markers With Cognitive Performance in South African HIV-Positive Patients. J. Neuroimmune Pharmacol. 14, 679–687. 10.1007/s11481-019-09870-1 31388873

[B125] Williams M. E. Joska J. A. Amod A. R. Paul R. H. Stein D. J. Ipser J. C. (2020). The Association of Peripheral Immune Markers With Brain Cortical Thickness and Surface Area in South African People Living with HIV. J. Neurovirol. 26, 908–919. 10.1007/s13365-020-00873-w 32661895

[B126] Winkler J. M. Chaudhuri A. D. Fox H. S. (2012). Translating the Brain Transcriptome in neuroAIDS: From Non-Human Primates to Humans. J. Neuroimmune Pharmacol. 7, 372–379. 10.1007/s11481-012-9344-5 22367717 PMC3354039

[B127] Woods S. P. Rippeth J. D. Frol A. B. Levy J. K. Ryan E. Soukup V. M. (2004). Interrater Reliability of Clinical Ratings and Neurocognitive Diagnoses in HIV. J. Clin. Exp. Neuropsychol. 26, 759–778. 10.1080/13803390490509565 15370374

[B128] Wright E. J. Grund B. Robertson K. Brew B. J. Roediger M. Bain M. P. (2010). I.S.S. Group, Cardiovascular Risk Factors Associated With Lower Baseline Cognitive Performance in HIV-Positive Persons. Neurology. 75, 864–873. 10.1212/wnl.0b013e3181f11bd8 20702792 PMC2938971

[B129] Xu Z. Asahchop E. L. Branton W. G. Gelman B. B. Power C. Hobman T. C. (2017). MicroRNAs Upregulated During HIV Infection Target Peroxisome Biogenesis Factors: Implications for Virus Biology, Disease Mechanisms and Neuropathology. Plos Pathog. 13, e1006360. 10.1371/journal.ppat.1006360 28594894 PMC5464672

[B130] Yelamanchili S. V. Chaudhuri A. D. Chen L.-N. Xiong H. Fox H. S. (2010). MicroRNA-21 Dysregulates the Expression of MEF2C in Neurons in Monkey and Human SIV/HIV Neurological Disease. Cell Death Dis. 1, e77. 10.1038/cddis.2010.56 21170291 PMC3002786

[B131] Yuan N. Y. Kaul M. (2021). Beneficial and Adverse Effects of cART Affect Neurocognitive Function in HIV-1 Infection: Balancing Viral Suppression Against Neuronal Stress and Injury. J. Neuroimmune Pharmacol. 16, 90–112. 10.1007/s11481-019-09868-9 31385157 PMC7233291

[B132] Zhou L. Pupo G. M. Gupta P. Liu B. Tran S. L. Rahme R. (2012). A Parallel Genome-Wide mRNA and microRNA Profiling of the Frontal Cortex of HIV Patients With and Without HIV-Associated Dementia Shows the Role of Axon Guidance and Downstream Pathways in HIV-Mediated Neurodegeneration. BMC Genomics. 13, 677. 10.1186/1471-2164-13-677 23190615 PMC3560210

